# Supplementary material for: A Common Variant in the Adaptor Mal Regulates Interferon Gamma Signaling
Source: Immunity. 2016 Feb 16;44(2):368–79. doi: 10.1016/j.immuni.2016.01.019 (PMC4760121; doi:10.1016/j.immuni.2016.01.019)
Supplement: Document S1. Figures S1–S6 and Supplemental Experimental Procedures [file mmc1.pdf]

Immunity, Volume *44*

Supplemental Information

A Common Variant in the Adaptor Mal

Regulates Interferon Gamma Signaling

Clíona Ní Cheallaigh, Frederick J. Sheedy, James Harris, Natalia Muñoz-Wolf, Jinhee Lee, Kim West, Eva Palsson McDermott, Alicia Smyth, Laura E. Gleeson, Michelle Coleman, Nuria Martinez, Claire H.A. Hearnden, Graham A. Tynan, Elizabeth C. Carroll, Sarah A. Jones, Sinéad C. Corr, Nicholas J. Bernard, Mark M. Hughes, Sarah E. Corcoran, Mary O'Sullivan, Ciara M. Fallon, Hardy Kornfeld, Douglas Golenbock, Stephen V. Gordon, Luke A.J. O'Neill, Ed C. Lavelle, and Joseph Keane

A.

```
human Mal   MASSTSLPAPGSRPKKPLGKMADWFRQTLLKKPKKRPNSPESTSSDASQP 50
murine Mal  MASSSSVPASSTPSKKPRDKIADWFRQALLKKPKKMPISQESHLYDGSQT 50
            ****:*:**..: .*** .*:******:******* * * **   *.**.

human Mal   TSQD--SP----------------LPPSLSSVTSPSLPPTHASDSGSS-- 80
murine Mal  ATQDGLSPSSCSSPPSHSSPESRSSPSSCSSGMSPTSPPTHVDSSSSSSG 100
            ::**  **                 *.* **  **: ****...*.**

human Mal   RWSKDYDVCVCHSEEDLVAAQDLVSYLEGSTASLRCFLQLRDATPGGAIV 130
murine Mal  RWSKDYDVCVCHSEEDLEAAQELVSYLEGSQASLRCFLQLRDAAPGGAIV 150
            ***************** ***:******** ************:******

human Mal   SELCQALSSSHCRVLLITPGFLQDPWCKYQMLQALTEAPGAEGCTIPLLS 180
murine Mal  SELCQALSRSHCRALLITPGFLRDPWCKYQMLQALTEAPASEGCTIPLLS 200
            ******** ****.********:****************.:*********

human Mal   GLSRAAYPPELRFMYYVDGRGPDGGFRQVKEAVMRYLQTLSWHLLYHGTP 230
murine Mal  GLSRAAYPPELRFMYYVDGRGKDGGFYQVKEAVIHYLETLS--------- 241
            ********************* **** ******::**:***

human Mal   EIGVKLETENPCRASDSHKCDKRYRE 256
            --------------------------
```

B.

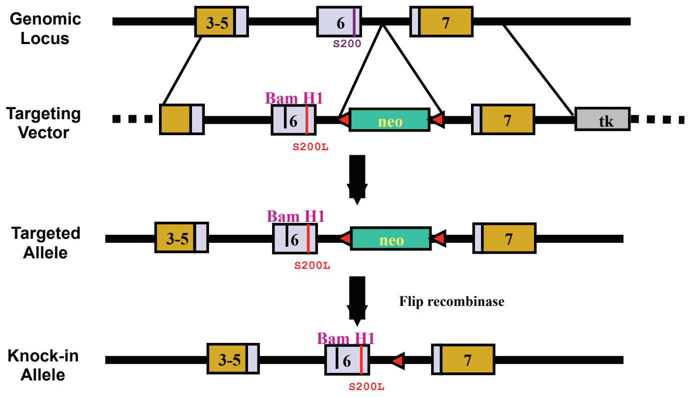

C.

Tirap-180:	human	mouse
WT allele	TCG=(Ser)	TCC=(Ser)
mutant allele	TTG=(Leu)	CTC=(Leu)

D.

E6

ACTGGTTCAGGCAGGCTCTGTTGAAGAAGCCCAAGAAGATGCCGATCTCCCAGGAAAGCC
ACCTCTATGATGGTTCACAGACAGCCACACAGGATGGTCTCTCACCCTCGAGCTGCAGCT
CACCCCCGAGTCACAGTTCACCGGAGAGCCGTAGCTCACCCTCGAGCTGCAGTTCAGGAA
TGTCACCTACCTCGCCACCAACACACGTGGACAGCAGCAGCAGCAGCAGTGGCCGCTGGA
GCAAAGACTACGATGTCTGCGTGTGCCACAGTGAGGAGGACTTGGAGGCGGCCCAGGAGC
TGGTCTCCTACTTGGAGGG**atc**CCAGGCCAGTCTACGCTGCTTCCTGCAGCTTCGGGATG
CAGCCCCGGGTGGCGCCATTGTTTCGGAGCTATGCCAGGCACTGAGTCGTAGTCACTGCC
GTGTGCTGCTCATCACTCCAGGCTTCCTTCGGGACCCCTGGTGCAAGTACCAGATGCTGC
AGGCCCTGACGGAGGCCCCGGCGTCGGAGGGTTGCACCATACCCCTGCTG*ct*CGGCCTGT
CCAGAGCCGCCTATCCGCCGGAACTCCGATTCATGTACTATGTGGATGGCAGAGGCAAGG
ACGGAGGCTTTTACCAAGTCAAGGAGGCTGTTATACACT

Supplementary Figure 1 - Design and generation of mutant *Tirap* S200L mice, the equivalent mutation of human S180L. A) Clustal alignment of human and murine Mal protein sequences. Serine 180 in human *TIRAP* (top line) and the corresponding amino acid in the murine TIR domain, Serine 200 (bottom line), is highlighted by a red box. **B)** Cloning strategy for the generation of S200L knock-in mice. Targeting vector was constructed covering a sequence from exon 3-7 of murine *Tirap.* This modified sequenced contained a mutated BamH1 site in exon 6 (for use in confirmation of recombination in ES cells by southern blotting and restriction digestion) as well as the mutated coding sequence for murine *Tirap* at codon 200 (TCC (Serine) -> **CT**C (Leucine) (illustrated in **C.**). It also contained the neomycin resistance gene flanked by 2 Flp sites. After confirming homologous recombination occured through southern blotting and digestion of fragments, the neomycin cassette was removed by incubation with Flp recombinase. The resulting DNA was sequenced and annotated sequence for exon 6 is shown in **D**. The resulting S200L mice generated were bred with wild-type mice to generate different combintions of the 3 genotypes annotated as $Tirap^{200S/S}$ (wild-type, Serine/Serine homozygotes), $Tirap^{200S/L}$ (Serine/Leucine heterozygotes) and $Tirap^{200L/L}$ (knock-in, Leucine/Leucine homozygotes), for use in experiments.

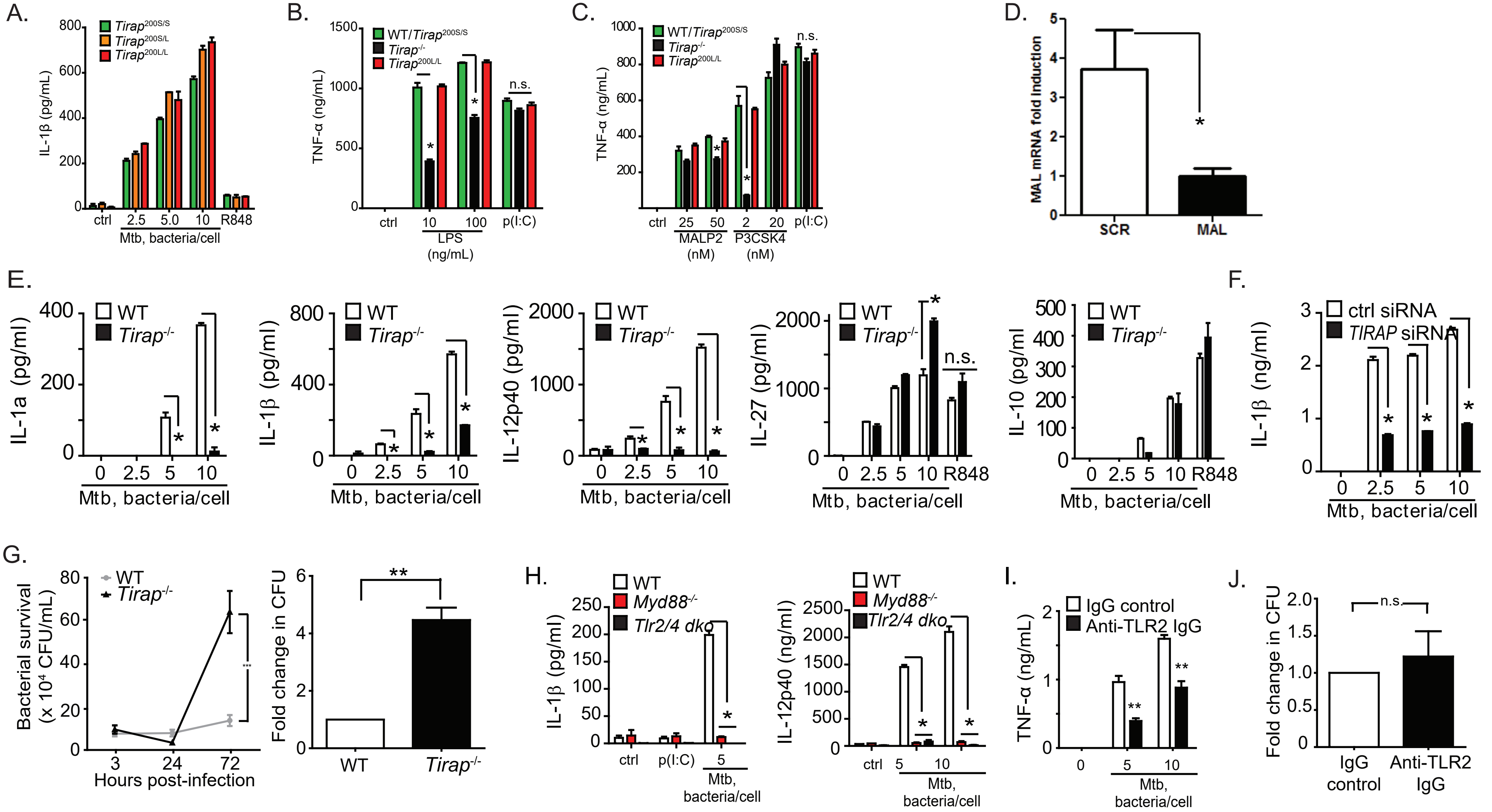

Supplemental Figure 2 - TLR signalling and *Mtb*-induced cytokine production is intact in murine S200L macrophages and Mal is required for cytokine production and killing of intracellular Mtb. (Figures 2 and 3)

(A) IL-1β secretion from primary BMM infected wth *Mtb* H37Rv at the indicated multiplicity of infection was measured in supernatants collected after 20 h stimulation and analysed by ELISA. **(B-C)** TNF-α secretion by primary BMM from wild-type (S/S), homozygote (L/L) and *Tirap*$^{-/-}$ mice stimulated for 20 h with LPS **(B)**, Malp-2, Pam3CysK4 (P3CSK4) or Poly I:C (p(I:C) - 100 ng/ml) **(C)**. All data is mean+/- S.D. from a single experiment (macrophages from 3 mice/group) representative of 3 independent experiments.

(D) confirmation of *TIRAP* knockdown by rtPCR. **(E)** Pro-inflammatory cytokine secretion by primary WT and *Tirap*$^{-/-}$ BMDM infected with Mtb H37Rv. **(F)** IL-1β secretion by THP-1 cells treated with *TIRAP* siRNA or scrambled control. **(G)** Primary murine BMM were infected with Mtb H37Rv and bacterial numbers determined. Data are means +/- S.D of data pooled from 3 separate experiments. **(H)** Pro-inflammatory cytokine secretion by WT, *Myd88*$^{/-}$ and *Tlr2/4* dko iBMM infection with Mtb H37Rv. **(I)** TNFα production by differentiated THP-1 treated with anti-TLR-2 antibody OPN305 or control IgG and infected with Mtb. Supernatants collected after overnight stimulation and analysed by ELISA for all experiments. **(J)** PMA-differentiated THP-1 cells treated with an anti-TLR2 antibody (OPN 305 1μg/ml) were infected with Mtb H37Rv and bacterial numbers determined as above. Data are means +/- S.D of data pooled from 3 separate experiments. * $p < 0.05$ (Mann-Whitney) for all experiments.

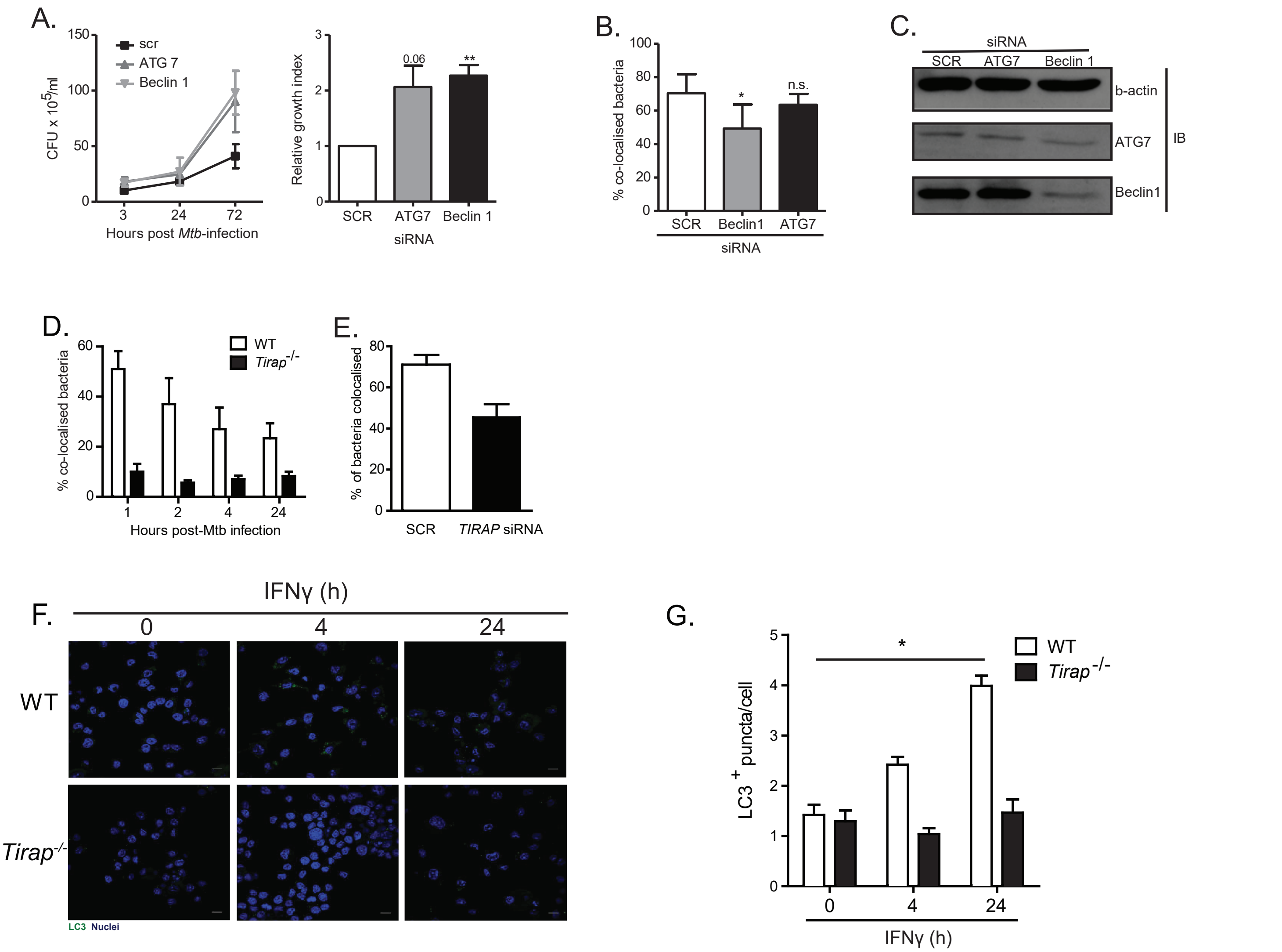

Supplemental Figure 3 - Autophagy deficient macrophages display defects in phagosome maturation and bactericidal activity similar to those seen in Mal-deficient macrophages. (A) WT iBMM were transfected with siRNA against *Beclin 1*, *ATG7* or scrambled control. Transfected iBMM were infected with *Mtb* and lysed at 3, 24 and 72 hours. Lysates were plated out for quantitation of cfus. Representative experiment (left hand panel) and means +/- SD of 3 pooled experiments (right hand panel). **(B)** Transfected iBMM were infected with FITC-labelled Mtb and stained with Lysotracker. Co-localisation was assessed by confocal microscopy. **(C)** Knockdown was assessed by immunoblotting. **(D)** BMDM from WT or *Tirap*$^{-/-}$ mice were infected with FITC-labelled *Mtb* for the indicated times between 0-24 h. Cells were stained with Lysotracker (LT). Co-localisation of *Mtb* with LT+ phagolysosomes was assessed by confocal microscopy. **(E)** THP-1 cells were transfected with siRNA against *Tirap* or scrambled control prior to differentiation with PMA. Cells were were stimulated overnight with recombinant human (rh)IFN-γ (20ng/ml) prior to being infection with FITC-labelled Mtb H37Rv. Cells were fixed at 2 h post-infection and stained with anti-LAMP-1 antibody and a fluorescent secondary antibody. Co-localisation of *Mtb* with LAMP-1+ phagolysosomes was assessed by with confocal microscopy. **(F-G)** Wild-type and *Tirap*$^{-/-}$ immortalised BMM were treated with rmIFN-γ for the time indicated. Cells were fixed, permeabilised and stained with fluorescently labelled anti-LC3 and analysed by confocal microscopy. Representative images shown in (F) and data quantified across 3 independent experiments and analysed by ANOVA (G).

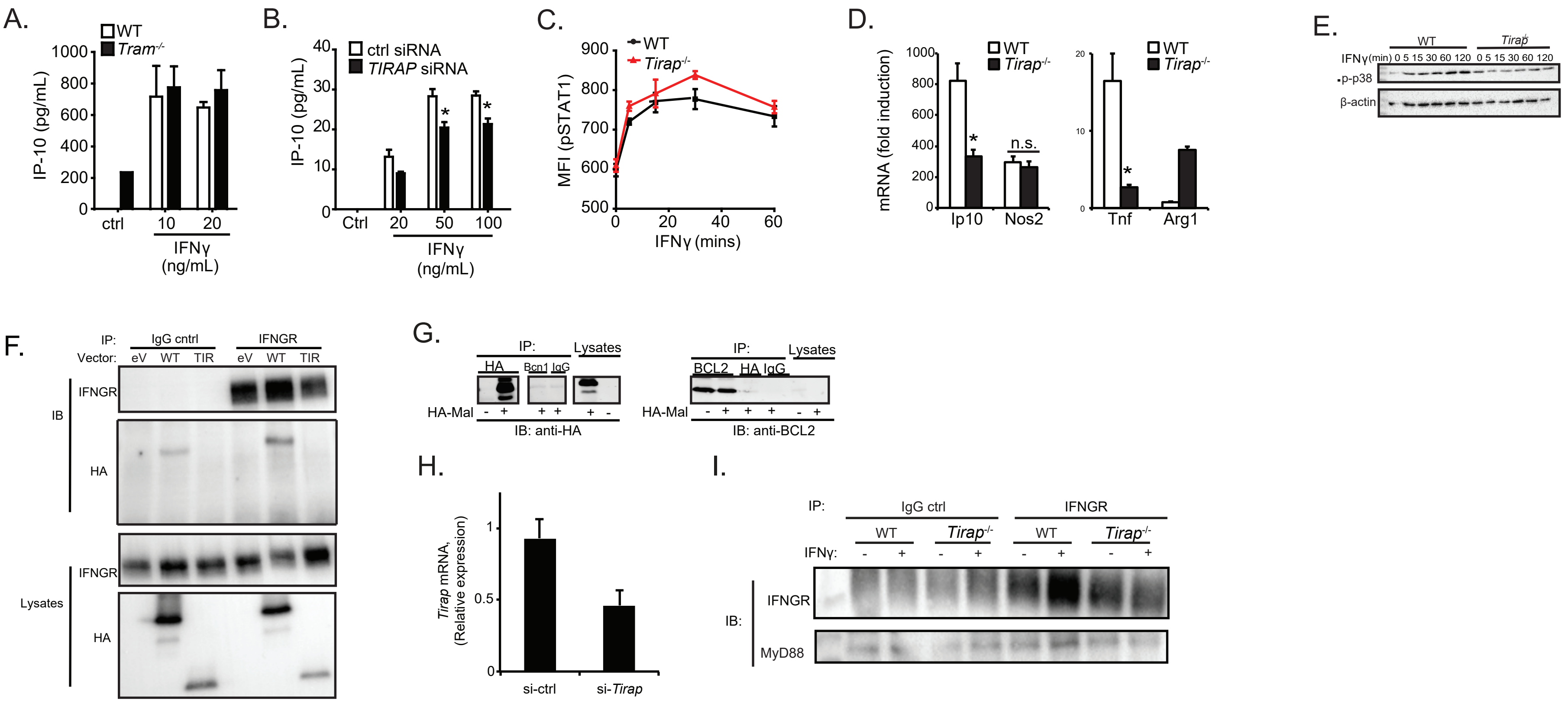

Supplementary Figure 5 - Mal independent processes in macrophage IFNγ signalling **(A)** WT and *Tram* -/- iBMM were treated with rmIFN-γ for 16 hours at the indicated concetrations and supernatants assessed for IP-10 secretion by ELISA. **(B)** THP-1 cells were transfected with siRNA against *TIRAP* or scrambled control prior to differentiation with PMA. Cells were were stimulated overnight with recombinant human (rh)IFN-γ (concentrations indicated) and supernatants analysed for IP-10 by ELISA. **(C)** iBMM were stimulated for the indicated times (0, 5, 15, 30, 60 min) with rmIFN-γ(10 ng/mL) before fixation and staining with anti-pSTAT1 (Y701) antibody conjugated to AlexaFluor 488 before analysis by flow cytometry using a BD FACSCanto II analyzer. **(D)** Primary BMDM (genotype indicated) were treated with recombinant IFN-γ at 20 ng/mL for 4h. RNA was extracted and gene expression analysed using qRT-PCR for the indicated genes. Data is relative fold incution over untreated cells and represents the mean +/- sd for triplicate determinations and is representative of 3 independent experiments. **(E)** Primary WT and *Tirap*-/- BMM were stimulated for for indicated times (0-2 h) with rmIFN-γ (20 ng/mL). Lysates were prepared and analyzed for phosphorylation of p38 MAP-kinase by immunoblotting with anti-p-p38 antibody (Cell Signaling, 9211 - top panel). Blots were stripped and re-probed for β-actin (bottom panel). Data shown are representative of 3 separate experiments. **(F)** HEK-293 cells were transfected with HA-tagged empty vector, HA-Mal or HA-TIR and immunoprecipitation was performed with antibodies to HA (Sigma, H6908) and IFNGR1 (Santa Cruz, sc-700), along with an IgG control, on cell lysates as indicated. Lysates were then blotted with anti-HA antibody. **(G)** HEK-293 cells were transfected with HA-tagged empty vector or HA-Mal and immunoprecipitation was performed with antibodies to HA (Sigma, H6908) and Beclin-1 or BCL-2, along with an IgG control, on cell lysates as indicated. Lysates were then blotted with anti-HA antibody. **(H)** RAW264.7 were transfected with small interfering RNAs specific to mouse *Tirap* (Ambion) for 72h. RNA was extracted and gene expression analysed using qRT-PCR for the indicated genes (*Tirap*) and is presented normalised to Gapdh and relative to untreated/untransfected cells. Data is mean +/- sd for triplicate determinations performed in parallel to the IP experiment shown in Fig 5E. **(I)** Primary WT and *Tirap* -/- BMM were stimulated for 2 h with rmIFN-γ (20 ng/mL) and immunoprecipitation was performed with antibodies to IFNGR1 or an IgG control on cell lysates as indicated. IP-samples were then analyzed for MyD88 expression by immunoblotting with anti-MyD88 (Millipore, 16527), alongside IFNGR1 expression. Data shown for all experiments are representative of 3 independent experiments. .

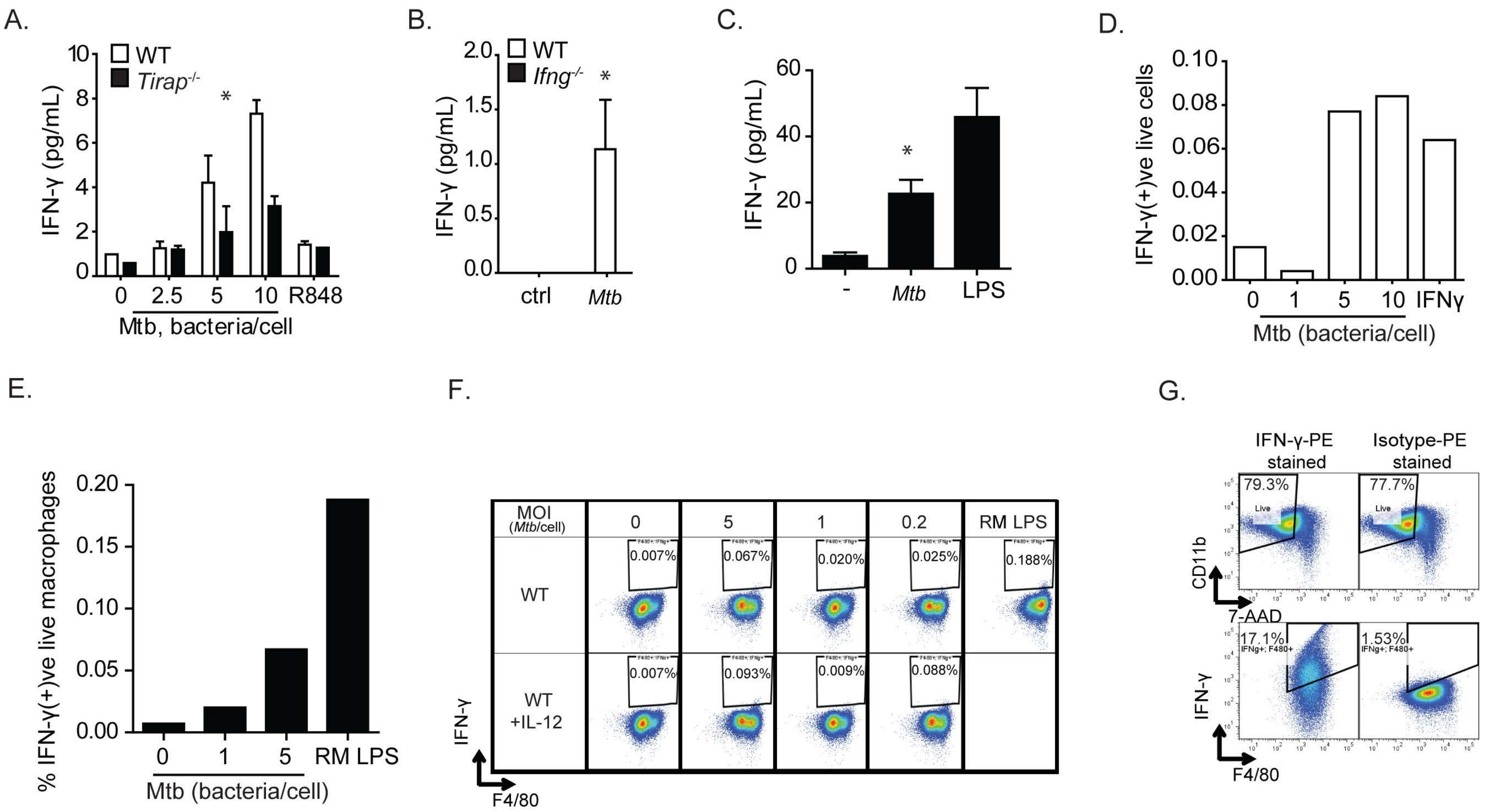

Supplemental Figure 5 - Interferon gamma production in infected macrophages. **(A-C)** Wildtype and *Tirap-/-* immortalized BMM **(A)** or wildtype and *Ifng-/-* primary BMMs **(B)** or PMA differentiated THP1 cells **(C)** were infected with *Mtb*. Supernatants were collected at 48 hours and analyzed for IFNγ production by ELISA. A two way ANOVA was used to compare cell types. Data shown are from a single experiment representative of 3 independent experiments. **(D)** Immortalized WT BMM were infected with *Mtb* for 72 hours. Cells were fixed, permeabilized and stained with live/dead Aqua and fluorescently labeled for the surface markers using anti-CD11b and anti-F4/80, and intracellular anti-IFNγ and analyzed by FACS. Data are expressed as frequency of IFNγ positive cells, gated on aqua negative (live) and CD11b+, F4/80+. Result shown is representative of 3 independent experiments. **(E and F)** Primary WT BMM were pretreated with IL-12 (3ng/ml; WT+IL-12) or medium control (WT) for 24 hours, and then infected with *Mtb* at the multiplicity of infection (MOI) indicated for 24 hours or tolerized with LPS as described in supplemental reference O'Carrol et al, 2013 for 48 hours. Cells were fixed, permeabilized and stained with intracellular fluorescently labeled anti-IFNγ and surface anti-F4/ 80and anti-CD11b and analyzed by FACS. Data representative of 3 independent experiments. **(G)** Cells were tolerized with LPS for 96 hours prior to restimulation with LPS as described in O'Carrol et al, 2013. Cells were fixed, permeabilized and stained with fluorescently labelled anti-IFNγ and anti-CD11b and anti-F4/ 80 and analyzed by FACS to assess intracellular production of IFNγ.

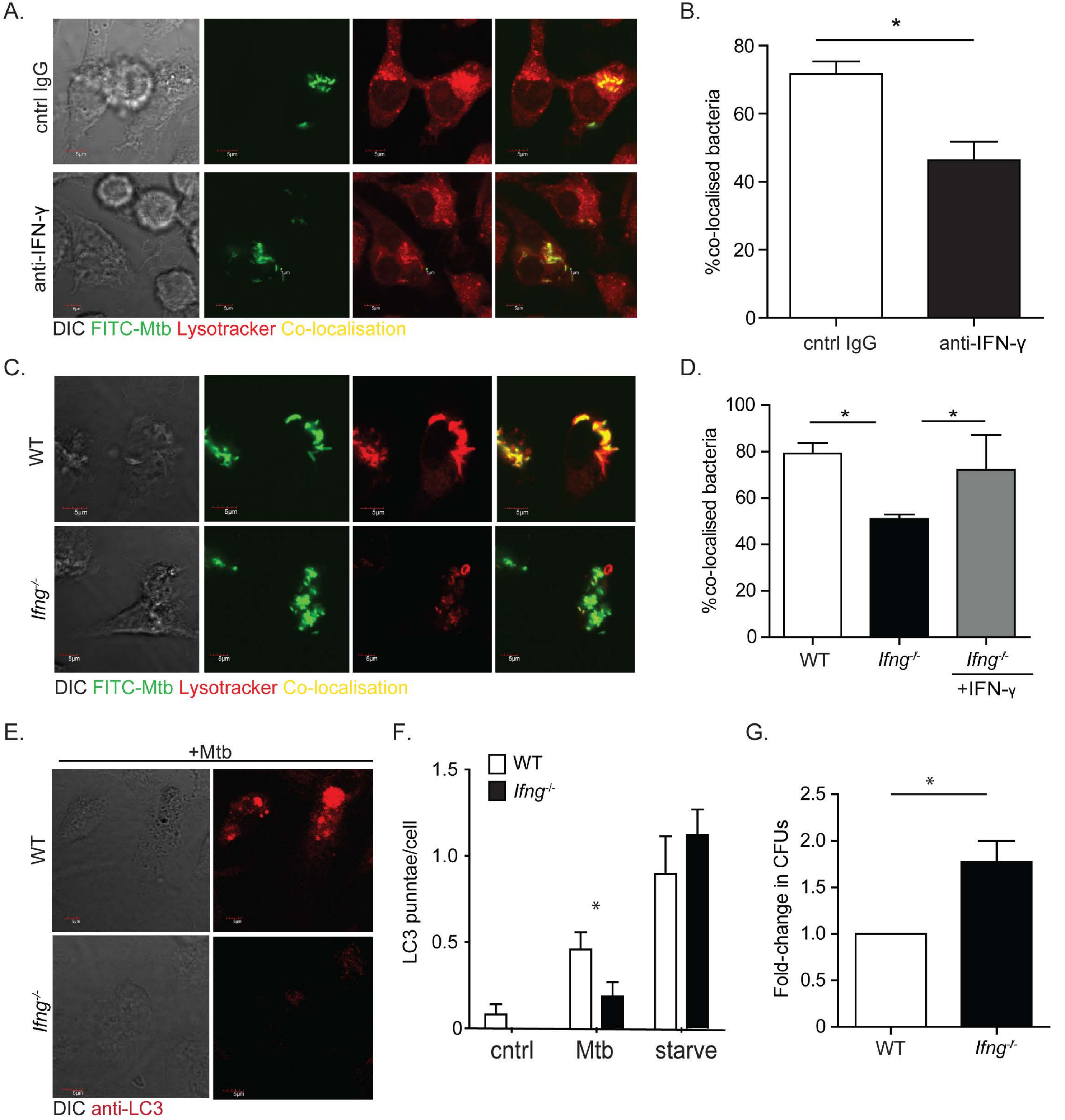

Supplemental Figure 6 - Interferon gamma production by *Mtb* infected macrophages has functional significance. (A and B) Immortalized WT BMM were pre-treated with anti-IFNγ antibody or IgG control prior to infection with FITC-labelled *Mtb*. Cells were stained with Lysotracker and fixed. Co-localisation was assessed by confocal microscopy. **(C and D)** Primary WT and *Ifng-/-* BMM with or without pre-treatment with IFNγ (20 ng/ml) were infected with FITC-labelled *Mtb*. Cells were stained with Lysotracker and fixed. Co-localisation was assessed by confocal microscopy. **(E and F)** Primary WT and *Ifng-/-* BMM were infected for 16 hours with *Mtb* or treated for 2 hours with starvation medium prior to fixation. Cells were stained with anti-LC3 antibody (Invitrogen) and LC3 puncta/cell were quantified by confocal microscopy. **(G)** Primary WT and *Ifng-/-* BMM were infected with *Mtb* and lysed at 72 h for quantification of bacteria. Experiments show mean +/- SD from a single experiment representative of 3 independent experiments and were analyzed by ANOVA.

Supplemental Experimental Procedures

Cell lines and culture

Primary bone-marrow derived macrophages were derived from the femurs of *Tirap*$^{-/-}$ (Mal knockouts), *Ifng*$^{-/-}$ and wildtype (WT) mice, and differentiated for 7 days with medium containing Macrophage-colony stimulating factor (M-CSF). THP-1 cells (ATCC) were transfected with siRNA against *TIRAP* (Mal) (Dharmacon) and scrambled control using the Amaxa nucleofector and reagents, prior to being differentiated into macrophage-like cells with phorbol myristate acetate (100nmol/L) for 24 h and then cultured in normal medium for a further 48 hours prior to stimulation.

Macrophage stimulation assays

Assessment of bacterial growth

Macrophages were grown at $1x10^5$ cells/ml in 12-well plates in antibiotic-free DMEM supplemented with 10% fetal calf serum. A suspension of *Mtb* H37Rv was prepared as above, and macrophages were infected with *Mtb* at a multiplicity of infection (MOI) of 10 bacteria/macrophage. Extracellular bacteria were washed off at 3 hours post-infection. Cells were lysed at the indicated time points by adding 0.1% Triton-X and scraping. Lysates were centrifuged at 3800 rpm to harvest bacteria, which were plated out at serial ten-fold dilutions on Middlebrook agar, and colonies were counted approximately 21 days later.

Phagosome maturation assays

Macrophages were grown on glass coverslips (Harris et al., 2008) and pretreated with IFN-γ or medium control overnight prior to infection. *Mtb* H37Rv was labeled with FITC (1mg/ml, Sigma) in accordance with the manufacturer's protocol. Bacteria were declumped with glass beads and sonicated prior to being put on to cells. Bacteria were incubated with cells for 1 hour, prior to washing and replacing medium for incubation for a further 1 hour. LysoTracker Red DND-99 (Invitrogen) at 100 nmol/L was added to macrophages one hour prior to fixation. Cells were fixed in 4% paraformaldehyde overnight. Alternatively, for CD63 staining, cells were permeabilized with 0.1% Triton X-100 in PBS after fixation, and blocked with 1% bovine serum albumin and 1% goat serum in PBS for 30 min at room temperature. Cells were incubated with primary antibody (mouse monoclonal antibody against CD63 /LAMP-3; Santa Cruz Biotechnology) at 1µg/mL for 1 h followed by secondary antibody (Alexa Fluor 488– or 568–labelled goat anti–mouse IgG (Invitrogen) were used at 4µg/mL), both at room temperature. Coverslips were mounted onto glass slides with fluorescent mounting medium (Dako), and images were recorded on an Olympus FluoView 1000 and a Zeiss LSM 510 laser scanning confocal microscope. Images were analyzed and prepared using the appropriate proprietary software.

Autophagy Analysis

Autophagosome formation was measured by LC3 punctate staining (Harris et al., 2001). Cells were treated as indicated, fixed using 4% paraformaldehyde, permeabilized using 0.1% Triton-X, blocked with 1% bovine serum albumin and 1% goat serum for 30 minutes at room temperature and incubated with primary antibody (LC3 antibody, Invitrogen) for 1 hour followed by secondary antibody (Alexafluor 488-labelled goat anti-rabbit IgG at 4µg/mL) for one hour. To quantify autophagy, fluorescence microscopy was used to count the number of

LC3- positive punctate dots in primary cells. Each condition was assayed in triplicate, and at least 100 cells per well were counted. Alternatively, LC3 conjugation in cell lysates was evaluated by Western blot analysis using an antibody raised to LC3-I/II (Sigma) used at 1/200, β-actin (Sigma); secondary antibodies (Licor) and chemiluminescence were used for detection. Autophagic flux was inhibited using either bafilomycin (100mM) or a combination of E64d and pepstatin.

Cytokine Measurements

Cytokine measurements were performed in supernatants using commercial ELISA kits from R&D Systems (murine IL-1α, IL-1β, IL-27, IP-10, IFNγ and TNF-α, and human IP-10), eBioscience (human TNF-α and IL-1β), Bioscience (murine IL-6, IL12p40 and IL-10) and Biosera (human IL-10)

Co-Immunoprecipitation

HEK293 cells were incubated for 24 h with DNA encoding various proteins in the presence of Genejuice. Cell lysates were prepared by lysis on ice for 10 min in 50 mM HEPES, pH 7.5, 250 mM NaCl, 20 mM βglycerophosphate, 1% Nonidet-P40, 2 mM dithiothreitol and a protease inhibitor 'cocktail' (Sigma) at a dilution of 1:500 for immunoprecipitation or by being boiled directly in Laemmli sample buffer for immunoblot. Immunoprecipitation was initiated by incubation of lysates for 2 h with protein A/G sepharose beads (Amersham) plus control antibodies. Precleared lysates were then incubated at 4 °C for at least 2 h with various antibodies and protein G beads (Amersham). Washed beads were boiled in sample buffer; proteins were separated by SDS-PAGE and were transferred onto nitrocellulose membrane. Membranes were blotted with the various antibodies and samples were visualized with an enhanced chemiluminescence system (Licor).

Plasmids

HA-Mal has been previously described (Valkov et al., 2011). Site-directed mutagensis was carried out to generate HA-tagged S180L variant Mal which was amplified using Miniprep (Qiagen). The sequences of both HA-Mal and HA-S180L Mal were confirmed by sequencing (Eurofins).

Fluorescence lifetime imaging microscopy-fluorescence resonance energy transfer (FLIM-FRET)

WT and *Tirap*$^{-/-}$ iBMM were treated with IFNγ (10 ng/ml) for 0, 0.5, 1, 2 and 24 h, then fixed in 2% paraformaldehyde and stained with antibodies against IFNGR2 (MyBioSource) and MyD88 (Abcam). For FLIM-FRET studies, they were secondary stained with Alexa Fluor A488 (donor) and A568 (acceptor) antibodies. An Olympus FV1000 microscope equipped with a PicoHarp300 FLIM extension and a 485 nm pulsed laser diode from PicoQuant was used to record FLIM data. Cells were first imaged by confocal microscopy using the Olympus FV1000 system to verify the presence of both donor and acceptor dye. Subsequently, corresponding FLIM images of donor fluorescence were recorded using the PicoHarp extension. Pixel integration time for FLIM images was kept at 40 μs per pixel and fluorescence lifetime histograms were accumulated to at least 10,000 counts in the maximum to ensure sufficient statistics for FLIM-FRET analysis. Photon count rates were kept below 5% of the laser repetition rate to prevent pileup.

Six FLIM images were recorded per time point, with 3 biological repeats per condition and 10 – 30 cells per image in the field of view. FLIM-FRET analysis was performed using the SymPhoTime 64 software (PicoQuant). The fluorescence lifetime decay curve for each image was summed over all pixels, then deconvolved with the measured instrument response

function (IRF) and fitted with a biexponential decay. The amplitude weighted average lifetime was extracted from each fit and averaged over all values of one sample condition. *p* values were determined to assess the significance of donor lifetime changes before and after treatment with IFNγ. A negative control (WT iBMM stained with IFNGR2 + A488 only) and positive control (WT iBMM stained with MYD88 + A488 + A568) were run alongside the samples.

Genotyping

Tirap genotype was determined on DNA extracted from buccal swabs (Isohelix, Cell Products). Ethical permission was granted by the Ethics Committee of the School of Medicine, Trinity College, Dublin. Informed consent was obtained. Genotyping of the Mal S180L and polymorphism was performed using the TaqMan Allelic Discrimination System (PE Biosystems, Foster City, California, USA). Genotyping results were verified using positive sequenced controls. PCR reactions were set up according to the manufacturer's instructions and thermal cycling was performed on 384-well reaction plates on PTC-225 DNA engine Tetrad (MJ Research, San Francisco, California, USA) as follows: initial denaturation and enzyme activation at 95°C for 10 min, followed by 40 cycles of denaturation at 95°C for 15 s and annealing/extension at 60°C for 60 s. Genotypes were determined using an ABI Prism 7900HT (PE Biosystems).

FACS Analysis

Immortalized macrophages were stimulated for the indicated times (0, 5, 15, 30, 60 min) with IFNγ at a final concentration of 10 ng/ml before washing and fixing in 1% PFA. Cells were then permeabilized with Permeabilization Wash Buffer (BioLegend) and stained with anti-pSTAT1 (Y701) antibody conjugated to AlexaFluor 488 at a dilution of 1/50 (Cell Signaling

Technology, clone 58D6) before analysis on a BD FACSCanto II analyzer. For analysis of intracellular IFNγ production primary or immortalized macrophages ($10x10^6$) were infected with the virulent *Mtb* strain at different multiplicities of infection for different times as indicated in the figures. Brefeldin A was added for 4 h at 40 μg/ml in the presence of *Mtb*. Supernatants were removed and detached cells were recovered by centrifugation at 400g for 5 minutes; remaining adherent cells were treated with lidocaine-HCl 4mg/ml supplemented with 10mM EDTA in $Ca2^{+}/Mg^{2+}$ free PBS for 15 min at 37ºC and detached by vigorous pipetting and finally pooled with the cells recovered previously. To assess purity of the monocultures, cells were stained with a PE-Cy7 labeled anti-mouse F4/80 (eBiosciences) and an APCefluor780 labeled anti-mouse CD11b (eBiosciences) in the presence of Fc block (BD). Dead cells were stained with 7AAD (eBiosciences) for 20 min and fixed with 2% PFA supplemented with actinomycin D (Sigma) at 40μg/ml to prevent background fluorescence due to 7AAD leakage from dead cells. Prior to intracellular staining, cells were permeabilized by incubation in PBS supplemented with 0.1% Saponin+ 0.1% BSA and then stained with a PE-labeled anti-mouse IFN-γ antibody (BD) or corresponding PE-labeled isotype control. *Mtb* was inactivated for 18 h by incubation of the cells in 4% PFA at 4ºC prior acquisition in a FACS Canto II equipped with 488, 633 and 405 lasers. One to two million cells were acquired on the live-cell gate per sample. The gating strategy was as follows: Single cells gated on FSC-H *vs* FCS-A >> Cells gated on FSC-A *vs* SSC-A >> Live (7AADneg), CD11b+ >> F4/80+, IFN-γ+. Gate for IFN-γ positive cells was determined by the corresponding FMO ("fluorescence minus one") control.

For LPS tolerization and recovery, macrophages were treated as previously described in (O'Carrol et al., 2013).

Mice

For *in vivo* experiments, pathogen-free male C57BL/6 (Jackson Laboratory) were maintained under barrier conditions in an animal room at University of Massachusetts Medical School. *Tirap* S200L heterozygote and homozygote mice (C57BL/6 background) were generated as described in Supplemental Figure 1. S200L mice were generated with C57BL/6 embryonic stem cells and C57BL/6 blastocysts. Animals were fed a sterile commercial mouse diet and water ad libitum. The University of Massachusetts Medical School Institutional Animal Care and Use Committee approved these experiments. A sample size for the *in vivo* experiment of 8 animals/group was chosen based on availability of mice. All mice were infected with *Mtb*, so randomisation was not appropriate. Mice were age and sex-matched, and were approimately 4 months old at the experimental endpoint. The investigator measuring the weight of the animals was blinded to the groups, otherwise no blinding was used.

Aliquots of frozen *Mtb* H37Rv were thawed and then sonicated for 5 minutes. A volume of sonicated stock previously titrated to deliver approximately 500 cfu per mouse was added to the nebulizer of a Glas-Col Inhalation Exposure System (Glas-Col, LLC, Terre Haute, IN) and mice were exposed to the infectious aerosol for 30 minutes (Martens et al., 2012). Mice were infected at approximately 8 weeks of age, and were kept in a pathogen-free BSL3 facility for a further 8 weeks. Two mice were killed 24 hours after infection to confirm the actual delivered dose.

Bacterial Load

At 8 weeks, mice were sacrificed. Lung homogenates from six mice were plated to measure bacterial burden. Lungs were homogenized in PBS containing 0.05% Tween-80, diluted serially 10-fold over 4 logs, and plated in duplicate on Middlebrook 7H11 agar (DIFCO,

Becton Dickinson, Sparks, MD). Plates were cultured at 37ºC for 3 weeks and then counted using a dissecting microscope to confirm colony morphology.

Lung Histology

Lungs were inflated and fixed with 10% buffered formalin for 24 hours and then processed for staining. Tissue sections were stained with hematoxylin and eosin (H&E). Sections were made at intervals spanning the whole lung, and the left cranial lobe was examined using light microscopy. Lung surface area of inflammation was measured with a Nikon Eclipse E400 microscope (Nikon Instruments, Melville, NY) at x 20 magnification using Spot Insight v3.5 software (Diagnostic Instruments Inc, Sterling Heights, MN). Percent total lung area involved with inflammation was calculated by dividing the cumulative area of inflammation by the total lung surface area examined for each lung studied.

Lung cytokine expression

Lungs were homogenized in PBS-T, an equal volume of cell lysis buffer (0.5% Triton X-100, 150 nM NaCl, 15 mM Tris, 1 mM $CaCl_2$ and 1mM $MgCl_2$, pH 7.4) was added and the mixture was vortexed, incubates (20 min, 4ºC), vortexed again, centrifuged (10 min, 12,000-14,000 x *g*) and the supernatant was filter-sterilized. Lung lysates were assayed for TNFα by ELISA (R&D Systems).

Statistical Analysis

A one-way ANOVA was performed to assess for statistically significant difference of the means between groups. Chi-squared analysis was used to assess statistically significant proportions of co-localization between groups. P values <0.05 were considered significant. Error bars represent standard deviations of the mean.

References

Harris, J., Hope, J.C., and Lavelle, E.C. (2009). Autophagy and the Immune Response to TB. Transboundary and Emerging Diseases 56 , 248-254.

O'Carroll, C., Fagan, A., Shanahan, F., Carmody, R. (2013). Identification of a Unique Hybrid Macrophage-Polarization State following Recovery from Lipopolysaccharide Tolerance. J. Immunol 192, 427-436.

Valkov, E., Stamp, A., Dimaio, F., Baker, D., Verstak, B., Roversi, P., Kellie, S., Sweet, M.J., Mansell, A., Gay, N.J., *et al.* (2011). Crystal structure of Toll-like receptor adaptor MAL/TIRAP reveals the molecular basis for signal transduction and disease protection. Proc Natl Acad Sci U S A *108*, 14879-14884.
