# Supplementary material for: A Common Variant in the Adaptor Mal Regulates Interferon Gamma Signaling
Source: Immunity. 2016 Feb 16;44(2):368–79. doi: 10.1016/j.immuni.2016.01.019 (PMC4760121; doi:10.1016/j.immuni.2016.01.019)
Supplement: Document S2. Article plus Supplemental Information [file mmc2.pdf]

Immunity

Article

A Common Variant in the Adaptor Mal Regulates Interferon Gamma Signaling

Graphical Abstract

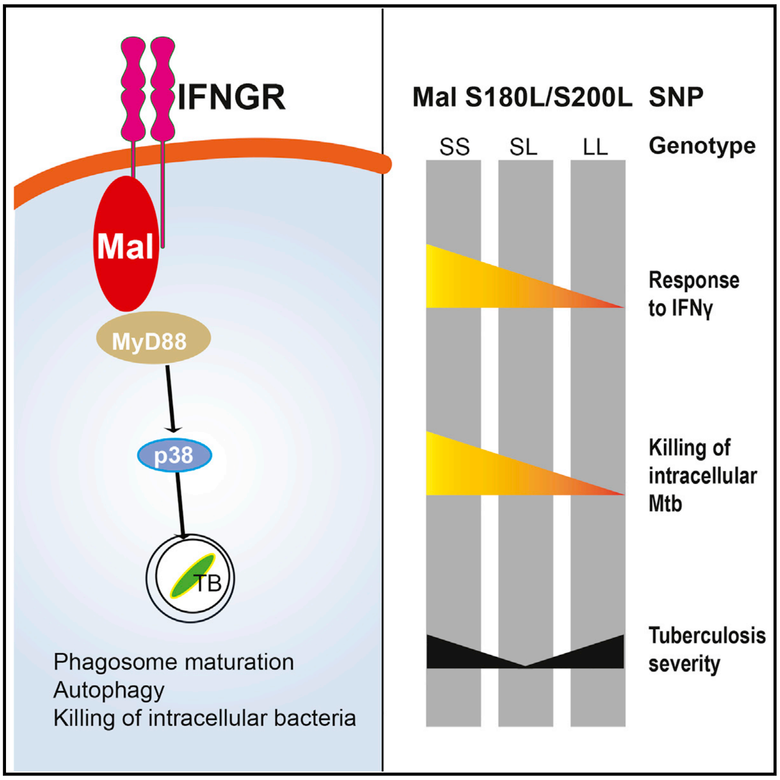

Highlights

- Mal has a TLR-independent role in IFNGR signaling
- IFNGR signaling via Mal leads to p38 phosphorylation, autophagy, and killing of TB
- The S180L mutation attenuates responses to IFN-γ stimulation
- S180L mutations impair in vitro and in vivo responses to TB

Authors

Clíona Ní Cheallaigh, Frederick J. Sheedy, James Harris, ..., Luke A.J. O'Neill, Ed C. Lavelle, Joseph Keane

Correspondence

nicheacm@tcd.ie (C.N.C.), lavellee@tcd.ie (E.C.L.)

In Brief

Mal (encoded by *TIRAP*) is a signaling adaptor in the TLR pathway. Ní Cheallaigh and colleagues demonstrate an additional role for Mal in IFN-γ signaling and find that it is required to kill intracellular *M. tuberculosis*. The common human Mal S180L polymorphism attenuates IFN-γ signaling and impairs responses to tuberculosis infection.

Ní Cheallaigh et al., 2016, Immunity *44*, 368–379
February 16, 2016 ©2016 The Authors
http://dx.doi.org/10.1016/j.immuni.2016.01.019

A Common Variant in the Adaptor Mal Regulates Interferon Gamma Signaling

Clíona Ní Cheallaigh,[1,2,*] Frederick J. Sheedy,[1] James Harris,[3] Natalia Muñoz-Wolf,[2] Jinhee Lee,[4] Kim West,[4] Eva Palsson McDermott,[5] Alicia Smyth,[6] Laura E. Gleeson,[1] Michelle Coleman,[1] Nuria Martinez,[4] Claire H.A. Hearnden,[2] Graham A. Tynan,[2] Elizabeth C. Carroll,[2] Sarah A. Jones,[3] Sinéad C. Corr,[5] Nicholas J. Bernard,[5] Mark M. Hughes,[5] Sarah E. Corcoran,[5] Mary O'Sullivan,[1] Ciara M. Fallon,[1] Hardy Kornfeld,[4] Douglas Golenbock,[4] Stephen V. Gordon,[6] Luke A.J. O'Neill,[5] Ed C. Lavelle,[2,7,8,*] and Joseph Keane[1,8]
[1]Department of Clinical Medicine, Institute of Molecular Medicine, Trinity College Dublin and St. James's Hospital, D08 W9RT, Dublin, Ireland
[2]Adjuvant Research Group, School of Biochemistry & Immunology, Trinity Biomedical Sciences Institute, Trinity College Dublin, D02 PN40, Dublin, Ireland
[3]Centre for Inflammatory Diseases, Southern Clinical School, Monash University Faculty of Medicine, Nursing and Health Sciences, Clayton, Victoria 3168, Australia
[4]Department of Medicine, University of Massachusetts Medical School, Worcester, MA 01655, USA
[5]Inflammation Research Group, School of Biochemistry & Immunology, Trinity Biomedical Sciences Institute, Trinity College Dublin, D02 PN40, Dublin, Ireland
[6]UCD Schools of Veterinary Medicine, Medicine and Medical Science, and Biomolecular and Biomedical Science, and UCD Conway Institute, University College Dublin, Belfield, Dublin 4, Ireland
[7]Advanced Materials and BioEngineering Research (AMBER), Centre for Research on Adaptive Nanostructures and Nanodevices (CRANN), Trinity College, D02 PN40, Dublin, Ireland
[8]Co-senior author
*Correspondence: nicheacm@tcd.ie (C.N.C.), lavellee@tcd.ie (E.C.L.)
http://dx.doi.org/10.1016/j.immuni.2016.01.019
This is an open access article under the CC BY license (http://creativecommons.org/licenses/by/4.0/).

SUMMARY

Humans that are heterozygous for the common S180L polymorphism in the Toll-like receptor (TLR) adaptor Mal (encoded by *TIRAP*) are protected from a number of infectious diseases, including tuberculosis (TB), whereas those homozygous for the allele are at increased risk. The reason for this difference in susceptibility is not clear. We report that Mal has a TLR-independent role in interferon-gamma (IFN-γ) receptor signaling. Mal-dependent IFN-γ receptor (IFNGR) signaling led to mitogen-activated protein kinase (MAPK) p38 phosphorylation and autophagy. IFN-γ signaling via Mal was required for phagosome maturation and killing of intracellular *Mycobacterium tuberculosis* (*Mtb*). The S180L polymorphism, and its murine equivalent S200L, reduced the affinity of Mal for the IFNGR, thereby compromising IFNGR signaling in macrophages and impairing responses to TB. Our findings highlight a role for Mal outside the TLR system and imply that genetic variation in *TIRAP* may be linked to other IFN-γ-related diseases including autoimmunity and cancer.

INTRODUCTION

Genetic variation in proteins involved in innate immunity, particularly Toll-like receptors (TLRs) and their signaling adaptor proteins, has been proposed to account for variation in susceptibility to infectious pathogens. MyD88 adaptor-like (Mal), encoded by the gene Toll-interleukin 1 receptor (TIR) domain-containing adaptor protein (*TIRAP*), was initially described as a signaling adaptor protein leading to nuclear factor kappa-light-chain-enhancer of activated B cells (NF-κB) activation downstream of TLR4 (Fitzgerald et al., 2001; Horng et al., 2001) and TLR2 (Horng et al., 2002; Yamamoto et al., 2002). A role for Mal as a "bridging adaptor" has since been established with Mal recruited to the plasma membrane, where it facilitates myeloid differentiation primary response gene 88 (MyD88) delivery to activated TLRs to initiate signal transduction in a structure called the Myddosome (Bonham et al., 2014; Kagan and Medzhitov, 2006). Mal has also been reported to function as a signaling adaptor for endosomal TLR signaling (Bonham et al., 2014).

Two non-synonymous single nucleotide polymorphisms (SNPs) in *TIRAP* with functional consequences have been identified, D96N and S180L. The S180L SNP is common in Indian and European (approximately 15%–20% carrying the L allele and 2%–3% being homozygous for the L allele) populations (Ferwerda et al., 2009) and results in alteration of a potential binding site near D96, leading to steric occlusion (Valkov et al., 2011). S180L has been associated with altered susceptibility to a number of infectious diseases including severe sepsis, severe pneumococcal disease, *Haemophilus influenzae,* and malaria (Ferwerda et al., 2009; Khor et al., 2007; Ladhani et al., 2010). An association has been reported between the S180L *TIRAP* polymorphism and TB susceptibility with heterozygotes for the mutation showing protection from disease and homozygotes showing increased susceptibility (Capparelli et al., 2013; Castiblanco et al., 2008; Khor et al., 2007; Selvaraj et al., 2010), although other studies have failed to replicate these findings (Dissanayeke et al., 2009; Nejentsev et al., 2008). A recent meta-analysis of the data confirms the association (Liu et al., 2014). The mechanism underlying the effect of the S180L SNP has not yet been elucidated.

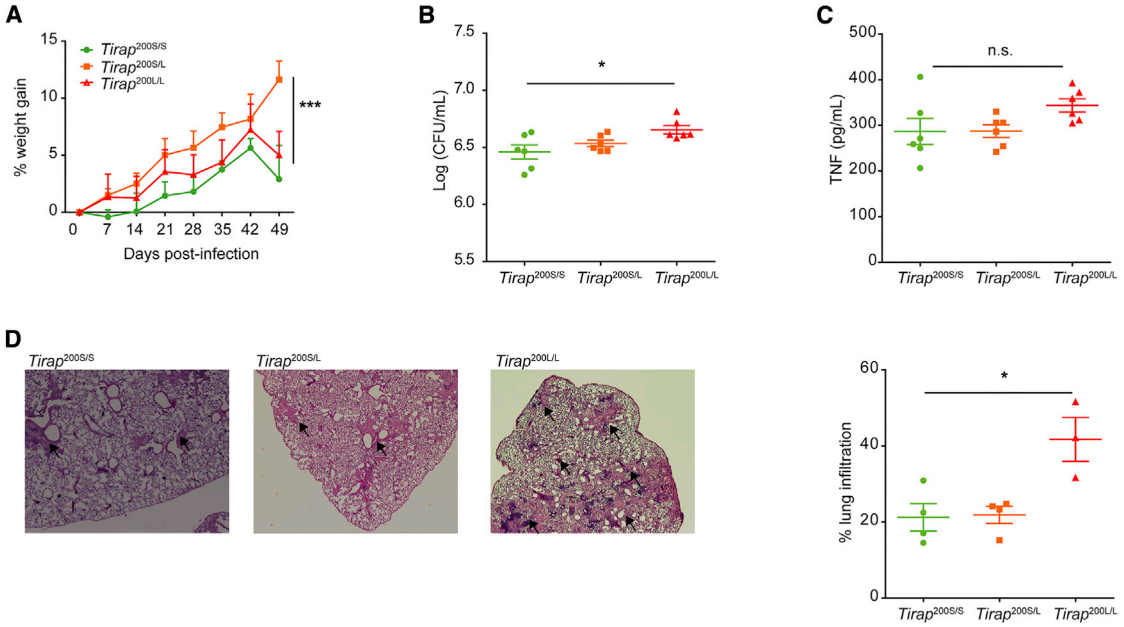

Figure 1. Mice Homozygous for *Mal* S200L, the Equivalent of *Mal* S180L, Develop More Severe Lung Inflammation in Response to In Vivo Infection with *Mycobacterium tuberculosis*

Age- and sex-matched WT ($Mal^{200S/S}$), heterozygote ($Mal^{200S/L}$), and homozygote ($Mal^{200L/L}$) mice were infected with 500 cfu of *Mycobacterium tuberculosis* (*Mtb*) H37Rv by aerosol.

(A) Weights of eight mice in each group were measured weekly.

(B) Mice were sacrificed at 8 weeks post-infection, and lung homogenates from five or six mice per group were plated for measurement of bacterial burden.

(C) TNF-α in lung homogenates from five or six mice per group was measured by ELISA.

(D) Lungs from three to four mice per group were fixed in formalin, stained with haemotoxylin and eosin, and area of inflammation assessed by microscopy with representative images and quantifications are shown. All data are means ± SD. A two-way ANOVA was used to analyze data in (A). A one-way ANOVA (non-parametric, Kruskal-Wallis) was used to analyze data in (B)–(D). *$p < 0.05$, ***$p < 0.001$) for all experiments.

Macrophages are key phagocytic cells that can eliminate or harbor intracellular bacteria, such as *Mtb* and also play a key role in secreting cytokines, which polarize subsequent adaptive immunity to a beneficial T helper 1 (Th1) or deleterious Th2 type response. Macrophages carry out a number of key antimicrobial functions including autophagy and phagosomal maturation, which if successful can kill intracellular mycobacteria (Deretic et al., 2006; Harris et al., 2009). IFN-γ plays a critical role in promoting antimicrobial functions. It activates macrophages, leading to production of nitric oxide (NO) and reactive oxygen species (ROS), phagosomal maturation, autophagy, and bactericidal activity (Gutierrez et al., 2004; MacMicking, 2012; Matsuzawa et al., 2014). Individuals with partial or complete defects in the IFN-γ signaling pathway have increased susceptibility to *Mtb*, as well as to other mycobacterial species (Bogunovic et al., 2012; Filipe-Santos et al., 2006).

In this study, we report that the murine equivalent of S180L, S200L, replicated human findings with homozygotes displaying increased severity of tuberculous disease. In vitro, the S200L mutation resulted in impaired phagosome maturation and killing of *Mtb*. We demonstrate that, unlike S180L, S200L did not affect TLR signaling. The impaired TB immunity seen with S200L was due to its effect on a Mal-dependent, TLR-independent, IFNGR signaling pathway. Mal-dependent responses to IFN-γ included p38 MAPK phosphorylation, autophagy, and phagosomal maturation, but not the canonical signal transducer and activator of transcription-1 (STAT-1) phosphorylation pathway. The S180L polymorphism reduced the affinity of Mal for the IFNGR, thereby compromising human macrophage responses to IFN-γ. This Mal-dependent IFNGR signaling pathway, modulated by the S180L mutation, might affect susceptibility to infectious diseases, inflammatory diseases, and cancer.

RESULTS

The S200L Mutation Is Associated with Increased Severity of TB Disease in Vivo

Mice with the equivalent of the human S180L mutation in *TIRAP* (*Tirap* 200L) were generated to provide an in vivo model of *Mtb* infection (Figure S1). Wild-type (SS), heterozygote (SL), and homozygote (LL) mice were infected with a high dose of *Mtb* H37Rv (a laboratory strain of virulent *Mtb)* via aerosol and weighed weekly. SL mice were protected against weight loss (Figure 1A). Mice were sacrificed at 8 weeks post-infection and lung lysates were analyzed. LL mice showed increased severity of TB disease, with an increase in bacterial burden (Figure 1B), despite similar levels of lung tumor necrosis factor alpha (TNF-α) production (Figure 1C). Most markedly however, we observed increased lung inflammation in LL mice (Figure 1D) compared to SL and SS mice. This correlates with the protection for heterozygotes and the increased susceptibility seen in homozygotes

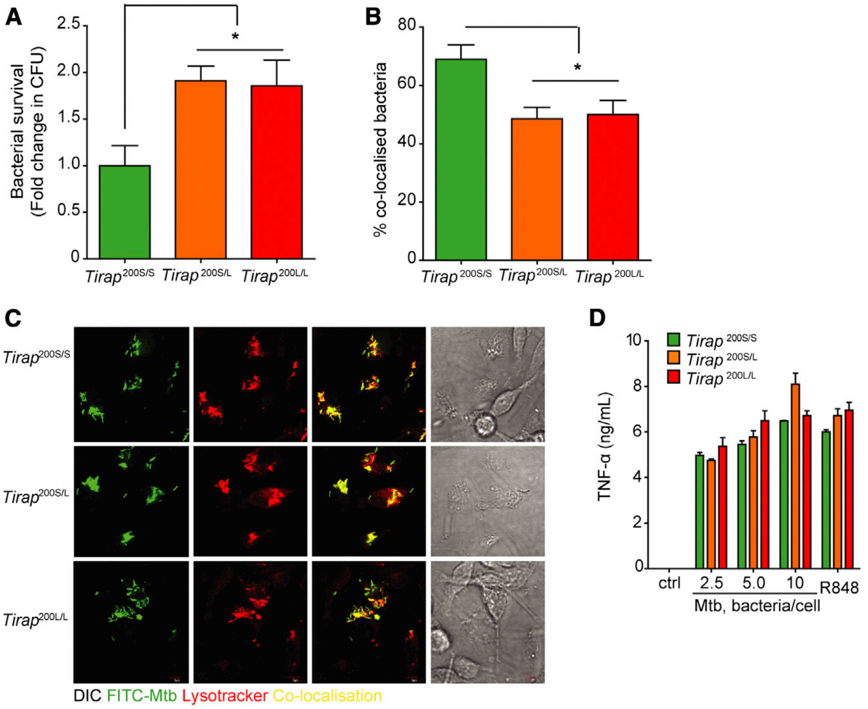

Figure 2. The S200L Mutation Impairs Macrophage Phagosome Maturation and Killing of Intracellular *Mtb*

(A) Primary BMM were infected with *Mtb* H37Rv and lysed at 72 hr. Serial dilutions of lysates were plated out to determine bacterial numbers.

(B and C) Cells were infected with FITC-stained *Mtb* H37Rv for 2 hr, fixed and stained with Lyso-tracker (LT, Life Technologies DND-99), and co-localization of *Mtb* with LT^+ phagolysosomes was assessed by confocal microscopy and quantified in (B) with representative images in (C).

(D) Primary BMM were infected overnight with *Mtb* H37Rv and supernatants analyzed for TNF-α production by ELISA. All experiments show mean ± SD pooled from three separate experiments with macrophages from one mouse per group in each experiment *p < 0.05 (one-way ANOVA used to analyze all experiments).

for the mutation in human studies. The phenotype of TB infection seen with the S200L mutation in mice replicated the human phenotype with S180L.

S200L Affects Macrophage Function Independently of Cytokine Production

Mycobacterial survival in macrophages is an in vitro read-out of macrophage function (Watson et al., 2012). Macrophages from mice with the S200L mutation displayed a defect in killing of intracellular mycobacteria (Figure 2A). Macrophages from mice with the S200L mutation displayed a defect in phagosome maturation, corresponding with the defect seen in bactericidal activity (Figures 2B and 2C). Unlike *Tirap*$^{-/-}$ macrophages described later, the S200L macrophages displayed no impairment in production of these cytokines (Figures 2D and S2A), indicating that the defect seen in mycobactericidal activity was not due to an impairment of cytokine production. In addition, S200L homozygote macrophages, unlike *Tirap*$^{-/-}$ macrophages, did not exhibit attenuated cytokine responses to TLR2 and TLR4 ligands (Figures S2B and S2C), indicating that S200L did not affect TLR2 or TLR4 signaling.

Mal and MyD88, but Not TLRs 2 and 4, Are Required for Macrophage Killing of Virulent Mtb

We proceeded to assess whether Mal-deficient macrophages showed a similar in vitro phenotype to S200L macrophages in response to *Mtb* infection. Immortalized and primary murine bone marrow macrophages were used, as well as *TIRAP*-silenced THP-1 cells. In contrast to our observations in S200L macrophages (Figure 2D), we observed that production of the key anti-mycobacterial cytokine TNF-α in response to *Mtb* was greatly reduced in Mal-deficient cells (Figures 3A–3C). Production of IL-1α, IL-1β, IL-6, and IL-12p40, but not IL-27 or IL-10, was also impaired in the absence of Mal (Figures S3A and S3B, with confirmation of *TIRAP silencing* in Figure S3C). Mal-deficient macrophages (Figures 3D and 3E and S3D) showed a marked inability to kill intracellular *Mtb*, similar to that seen in the S200L macrophages. We also assessed the role of TLRs 2 and 4 in our model. *Tlr2*$^{-/-}$, *Tlr2/4* double knockout (*dko)* and *Myd88*$^{-/-}$ macrophages replicated the defect in pro-inflammatory cytokine production seen in Mal deficient cells. *Tlr4*$^{-/-}$ macrophages showed a smaller, but still significant, impairment in cytokine responses (Figures 3F and 3G and S3E and S3F). Notably, whereas *Myd88*$^{-/-}$ macrophages replicated the defect in bactericidal activity seen in *Tirap*$^{-/-}$ cells, *Tlr2*$^{-/-}$, *Tlr4*$^{-/-}$, and *Tlr2/4 dko* murine macrophages and THP-1 macrophages treated with an anti-TLR2 antibody did not show a similar defect despite impairments in cytokine induction (Figures 3H and 3I and S3G). Consistent with our findings is a previous report of unimpaired bactericidal activity of *Tlr2/4/9* triple knockout macrophages and impaired restriction of *Mtb* growth by *Myd88*$^{-/-}$ macrophages (Hölscher et al., 2008). These data indicated that Mal and MyD88 had a function in killing of *Mtb*, and that this was distinct from the known role of Mal downstream of TLRs 2 and 4. This defect in mycobactericidal activity seen in the absence of Mal, but not in the absence of TLRs 2 and 4, was not due to defects in cytokine production.

Mal Is Required for Autophagy and IFN-γ Induced Phagosome Maturation

We sought to identify Mal-dependent but TLR-independent macrophage effector mechanisms. Autophagy provides a mechanism of killing and removing intracellular pathogens and contributes to a number of critical host immune responses to *Mtb* (Ní Cheallaigh et al., 2011). To determine whether *Mtb*-induced autophagic flux was impaired in the absence of Mal, we infected primary macrophages with *Mtb* in the presence or absence of bafilomycin, which blocks autophagosome fusion with lysosomes and thereby completion of autophagy and breakdown

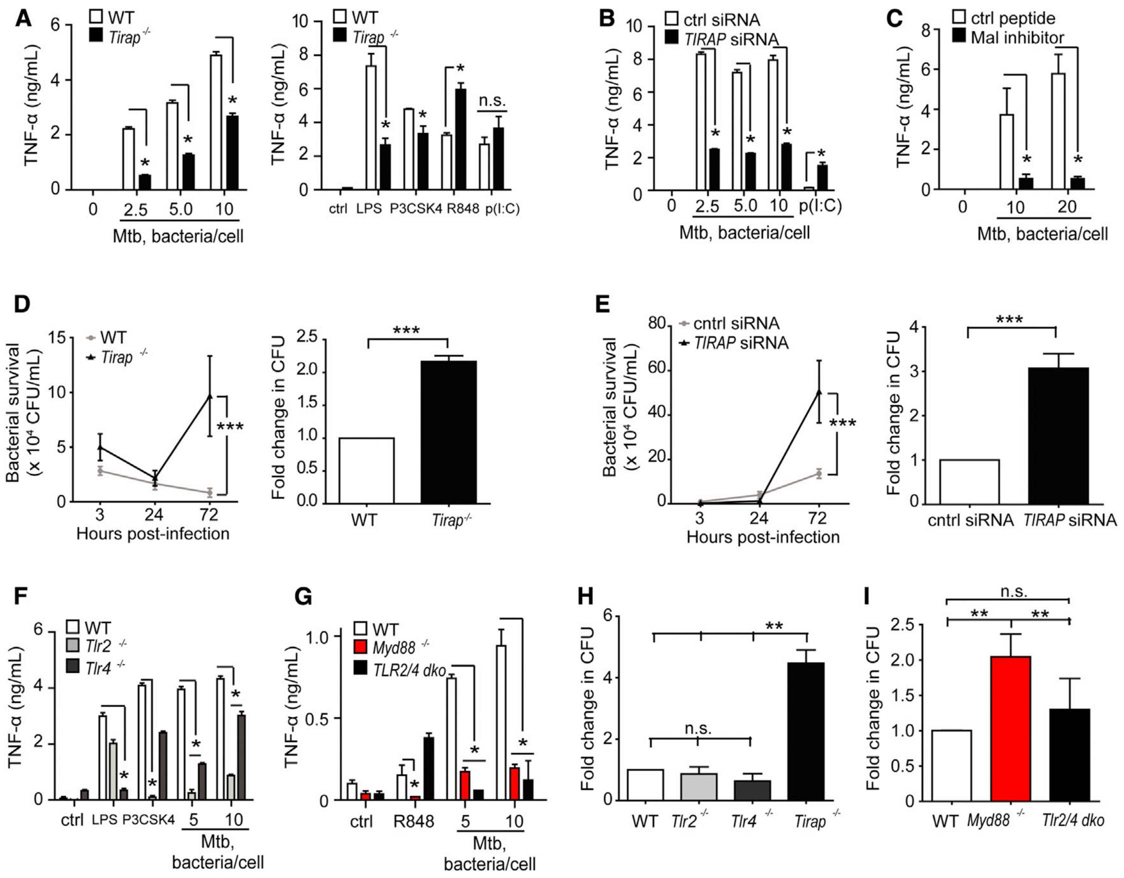

Figure 3. Mal and MyD88, but Not TLR2 or TLR4, Are Required for Macrophage Killing of Virulent *Mycobacterium tuberculosis*

(A) TNF-α secretion by murine wild-type (WT) and *Tirap*$^{-/-}$ immortalized bone marrow-derived macrophages (iBMM) (1 × 10^6/ml) infected with *Mtb* H37Rv (left panel) or treated with the indicated TLR ligands (right panel), was measured in supernatants collected after 20 hr stimulation and analyzed by ELISA.

(B) Cytokine secretion by PMA-differentiated THP-1 (5 × 10^5/ml) cells transfected with siRNA against Mal or scrambled control in response to infection with *Mtb* H37Rv (20 hr) was measured by ELISA.

(C) ELISA of TNF-α secretion by PMA-differentiated THP-1 cells (5 × 10^5/ml) treated with a Mal inhibitor peptide (TIRAP inhibitory peptide, Calbiochem, 613571) before infection with *Mtb* H37Rv (20 hr).

(D) WT and *Tirap*$^{-/-}$ iBMM were infected with *Mtb* H37Rv at a multiplicity of infection of 10 bacteria/cell and lysed at 3, 24, and 72 hr. Serial dilutions of lysates were plated out to determine bacterial numbers. Left panels show data representative of at least three separate experiments, right panels are mean ± SD pooled from bacterial counts at 72 hr from three separate experiments.

(E) PMA-differentiated THP-1 cells transfected with siRNA against *TIRAP* (Dharmacon/Thermoscientific) or scrambled control siRNA, were infected with *Mtb* H37Rv and bacterial numbers determined as above. Left panel shows data representative of at least three separate experiments, and right panel is mean ± SD pooled from three separate experiments.

(F and G) TNF-α secretion by iBMM (1 × 10^6/ml) infected with *Mtb* H37Rv (20 hr) was measured by ELISA.

(H and I) iBMM were infected with *Mtb* H37Rv and bacterial numbers determined as above. Data are means ± SD of data pooled from three separate experiments. *$p < 0.05$ (Mann-Whitney) for all experiments.

of autophagosomes (Yamamoto et al., 1998). *Mtb*-induced autophagic flux was reduced in the absence of Mal (Figure 4A-B). When autophagy was impaired in macrophages using knockdown of the key effector proteins ATG7 or BECLIN-1 by siRNA, a similar defect in bactericidal activity and phagosomal maturation to that seen in Mal-deficient cells was observed (Figures S4A–S4C).

We assessed whether Mal was required for phagosomal maturation by pretreating macrophages with IFN-γ or RPMI control and infecting the macrophages with FITC-labeled live *Mtb*. We identified phagolysosomes using anti-CD63 antibody or LysoTracker Red and assessed co-localization of bacteria and phagolysosomes. IFN-γ pre-treatment increased co-localization of bacteria and phagolysosomes in WT, but not in *Tirap*$^{-/-}$ macrophages (Figures 4C and 4D [LysoTracker] and Figures 4E and 4F [CD63]) a trend which persisted up to 24 hr post IFN-γ treatment (Figure S4D). Similar results were obtained in differentiated THP-1 cells treated with siRNA against Mal and stained with LysoTracker or the mature endolysosomal marker LAMP-1 (Figures 4G and S4E). Notably, IFN-γ did increase phagosomal maturation in *Tlr2/4* dko, but not *Myd88*$^{-/-}$ macrophages (Figure 4H). Thus, Mal and MyD88, but not TLRs 2 and 4, were required for IFN-γ-induced phagosome maturation.

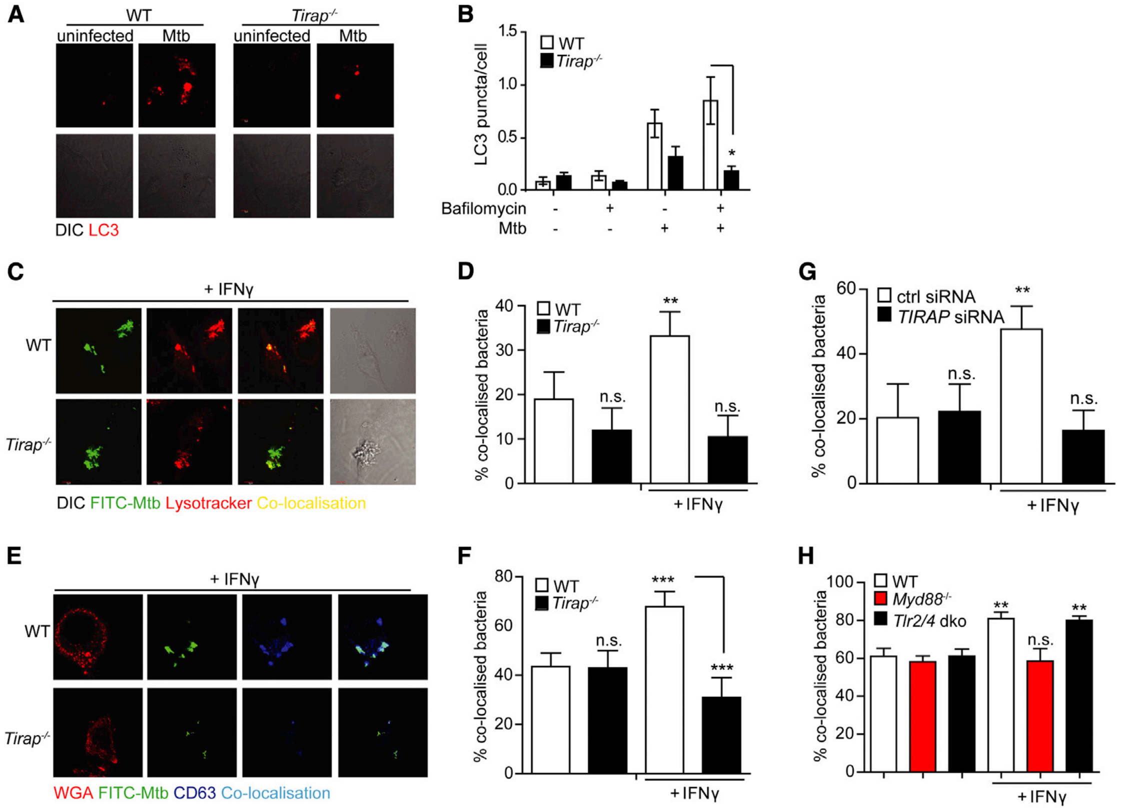

Figure 4. Mal Regulates IFN-γ Induced Maturation of *Mtb* Containing Phagosomes Independently of TLR2 and TLR4

(A and B) Primary WT and *Tirap*$^{-/-}$ bone-marrow-derived macrophages (BMM) were stimulated for 16 hr with *Mtb* in the presence or absence of bafilomycin. Cells were stained with anti-LC3 antibody (Invitrogen L10352) (representative images in (A) and LC3^{+} puncta per cell quantified by confocal microscopy (B).

(C–F) WT and *Tirap*$^{-/-}$ iBMM were stimulated overnight with rmIFN-γ (20 ng/ml) prior to infection with FITC-labeled *Mtb* H37Rv. Cells were stained with Lysotracker (LT) (C and D) and co-localization of *Mtb* with LT^{+} phagolysosomes was assessed by confocal microscopy, (representative images in C, quantified in D). Alternatively, cells were stained with anti-CD63 antibody (Santa Cruz, H-193) (E and F) and co-localization of *Mtb* with CD63-positive phagolysosomes was assessed by confocal microscopy, (representative images in E, quantified in F).

(G) THP-1 cells were transfected with siRNA against *TIRAP* or scrambled control prior to differentiation with PMA. Cells were stimulated overnight with recombinant human (rh)IFN-γ (20 ng/ml) prior to infection with FITC-labeled *Mtb* H37Rv and stained with LT. Co-localization of *Mtb* with LT^{+} phagolysosomes was quantified by confocal microscopy.

(H) iBMM were infected with FITC-labeled *Mtb* H37Rv and stained with LT. Co-localization of *Mtb* with LT^{+} phagolysosomes was quantified by confocal microscopy. Data shown are mean ± SD from a single experiment representative of three separate experiments are shown for (B) and mean ± SD of data pooled from three separate experiments for all other experiments (D, F–H). *$p < 0.05$ (Mann-Whitney) for all experiments.

Mal and MyD88, but Not TLRs 2 and 4, Are Required for Interferon-Inducible Protein 10 Production, p38 Phosphorylation, and Autophagy in Response to IFN-γ

Given our findings of impaired IFN-γ induced phagosomal maturation in the absence of Mal and MyD88 but not TLRs 2 and 4, we hypothesized that the Mal-dependent but TLR-independent pathway required for killing of intracellular *Mtb* might be explained by Mal participating in MyD88-dependent IFNGR signaling. Previously, normal STAT-1 phosphorylation but impaired IFN-inducible protein 10 (IP-10) secretion was reported in MyD88-deficient macrophages (Sun and Ding, 2006). We identified a profound defect in IFN-γ-induced IP-10 secretion in *Tirap*$^{-/-}$ and Myd88$^{-/-}$ macrophages, but not in *Tlr2/4* dko macrophages or *Tram*$^{-/-}$ macrophages (Figure 5A and S5A). IP-10 secretion was also reduced in *TIRAP*-silenced THP-1 cells (Figure S5B). IFN-γ-induced STAT-1 phosphorylation remained intact in Mal-deficient cells (Figure S5C). We observed a reduction in *Cxcl10/Ip10* mRNA and *Tnfa* mRNA in *Tirap*$^{-/-}$ macrophages after IFN-γ treatment—a trend not observed for the STAT-1 target gene, *Nos2*, or for *Arg1* mRNA (Figure S5D).

P3 mitogen-activated protein kinases (MAPK) are a class of MAPK that respond to stress stimuli including cytokines and are involved in apoptosis and autophagy. An IFNγ-induced p38 MAPK signaling pathway, which culminates in autophagy and killing of intracellular bacteria, was recently reported (Matsuzawa et al., 2014). We hypothesized that Mal might be required for this pathway. P38 MAPK was phosphorylated in response to IFN-γ, peaking at 4 hr post-treatment (Figures 5B, top panel), and this was impaired in *Tirap*$^{-/-}$ macrophages (Figure 5B, bottom panel and S5E). IFN-γ induced autophagy, but not starvation-induced autophagy, was reduced in *Tirap*$^{-/-}$ macrophages, (Figures 5C and S6A and S6B). These data demonstrate that Mal was required for IFN-γ induced P38 MAPK phosphorylation, autophagy and IP-10 secretion.

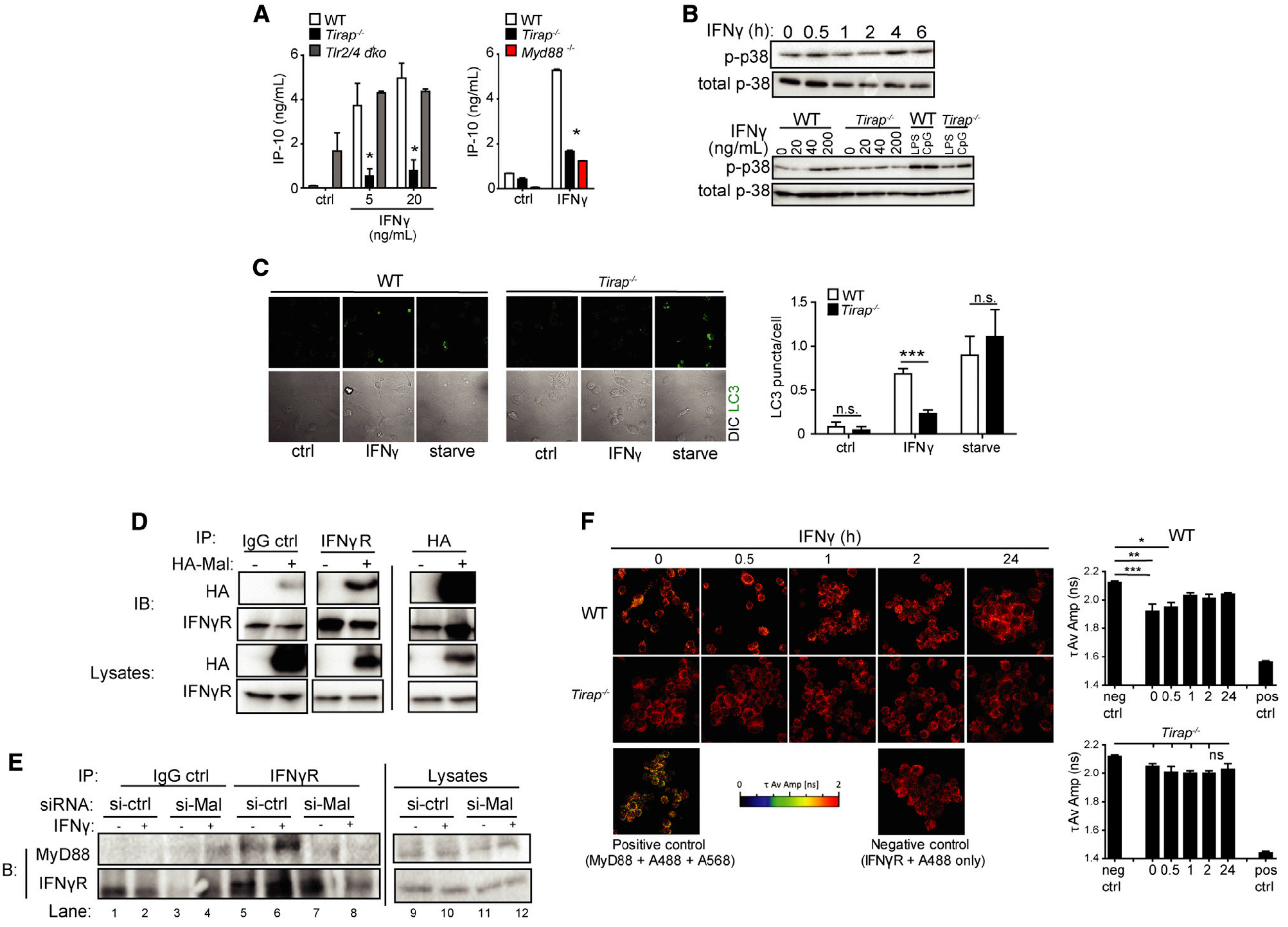

Figure 5. Mal Associates with the IFN-γR and Is Required for IFN-γ Induced p38 Phosphorylation and Autophagy

(A) iBMM were treated with rmIFN-γ for 20 hr and secretion of IP-10 was measured by ELISA. Data are means ± SD from a single experiment representative of three separate experiments $*p < 0.05$, (Mann-Whitney).

(B) Primary BMM were treated for the indicated times (0–6 hr) at 20 ng/mL (top panel) and for 4 hr with rmIFN-γ at the indicated concentrations (0–100 ng/mL, bottom panel) alongside LPS or R848 (10 min). Lysates were prepared and analyzed for phosphorylation of p38 MAP-kinase by immunoblotting with anti-p-p38 antibody (Cell Signaling, 9211). Blots were stripped and re-probed for total p38 (bottom panels). Data shown are representative of three separate experiments.

(C) Primary BMM were stimulated for 16 hr with rmIFN-γ (20 ng/ml) and bafilomycin (100nM) or for 2 hr with starvation medium. Cells were stained with anti-LC3 antibody and LC3 puncta per cell were quantified with confocal microscopy. Data are means ± SD from a single experiment representative of three separate experiments, $*p < 0.05$, (Mann-Whitney).

(D) HEK293 cells were transfected with HA-tagged Mal and immunoprecipitation was performed with antibodies to HA (Sigma, H6908) and interferon gamma receptor (Santa Cruz, sc-700), along with a control non-specific rabbit IgG, on cell lysates as indicated. Lysates were then blotted with anti-HA antibody. Data shown are representative of three independent experiments.

(E) RAW264.7 cells were transfected with the indicated siRNAs (50 nM) for 72 hr prior to treatment with IFN-γ (100 ng/ml, 10 min) and immunoprecipitation was performed with antibodies to IFN-γR1 or an IgG control on cell lysates as indicated. IP-samples were then analyzed for MyD88 expression by immunoblotting with anti-MyD88 (Millipore, 16527) (top panel) alongside IFN-γR1 expression (bottom panels). Data shown are representative of three independent experiments.

(F) iBMM were treated with IFN-γ (10 ng/ml) for 0–24 hr. Cells were fixed and stained with antibodies against MyD88 and IFNGR2 followed by fluorescent secondary antibodies (Alexa Fluor 568 and Alexa Fluor 488). Changes in the amplitude weighted average lifetime (τ Av Amp) of the donor (A488) due to proximity with the acceptor (A568) were measured. A decrease in τ Av Amp indicates interaction between the molecules and is quantified on right. $*p < 0.05$, $**p < 0.01$, $***p < 0.005$; one-way ANOVA.

Mal Interacts Directly with the IFNGR

We hypothesized that Mal might act as a bridging adaptor for MyD88 and the IFNGR. Immunoprecipitation of endogenous IFNGR resulted in co-immunoprecipitation of overexpressed Mal (Figure 5D, left panel). This interaction required full-length Mal, as a mutant construct consisting solely of the TIR domain did not immunoprecipitate with the IFNGR (Figure S7A). Immunoprecipitation of full-length overexpressed HA-Mal resulted in co-immunoprecipitation of IFNGR, confirming that Mal can bind IFNGR directly (Figure 5D, right panel). Mal did not precipitate with other proteins including Beclin-1 and BCL-2 (Figure S7B).

We then hypothesized that if Mal functions as a bridging adaptor between MyD88 and the IFNGR, then the previously

reported interaction between MyD88 and the IFNGR (Sun and Ding, 2006) would be reduced in the absence of Mal. We observed increased recruitment of MyD88 to IFNGR in IFNγ-treated control cells (Figure 5E, lanes 5/6); however, in *TIRAP*-silenced or Mal-deficient cells (knockdown shown in Figure S7C) this interaction was reduced to background levels (Figure 5E, lanes 7/8 and Figure S7D).

We then confirmed this finding using fluorescence lifetime imaging microscopy-fluorescence resonance energy transfer (FLIM FRET) technology. FRET occurred between IFNGR and MyD88, indicating that they are interacting basally and for up to 0.5 hr post IFN-γ treatment (Figure 5F) in WT cells. This steady-state interaction between MyD88 and IFNGR was disrupted after initial signal transduction. In *Tirap*$^{-/-}$ cells, there was no evidence of such a basal interaction, a pattern not altered by subsequent IFN-γ treatment. These data demonstrated that Mal bound to IFNGR and was required for the interaction between MyD88 and IFNGR.

Interferon Gamma Is Required for Autophagy and Phagosome Maturation in Response to Mtb

Our data had established that Mal is required for an IFN-γ signaling pathway, culminating in p38 MAPK phosphorylation and autophagy. We hypothesized that the observed defect in autophagy and killing of intracellular *Mtb* was due to the defect in the IFN-γ induced p38 MAPK phosphorylation pathway. However, we observed the deficits in autophagy and killing in a monoculture of macrophages in the absence of exogenous IFN-γ. We therefore hypothesized that macrophages secrete small quantities of IFN-γ that are functionally relevant in our model. Immortalized and primary macrophages and differentiated THP-1 cells produced small but detectable amounts of IFN-γ when infected with *Mtb* (Figures S8A–S8C). IFN-γ production was reduced in *Tirap*$^{-/-}$ immortalized bone-marrow-derived macrophages (iBMM) (Figure S8A)—this may be due to impaired TLR2 and/or TLR4 signaling or impaired IFN-γ signaling, because IFN-γ can upregulate its own production in a positive feedback loop. We also observed IFN-γ production by immortalized and primary macrophages using intracellular staining and flow cytometry (Figures S8D–S8F). Macrophages produced a considerable amount of IFN-γ if allowed to recover from LPS tolerization and restimulated for 4 hr (Figures S8E–S8G). Although our data show that *Mtb* can induce IFN-γ production by macrophages, the levels produced are extremely low compared to those produced by T cells or NK cells. We therefore sought to assess whether the low levels of IFN-γ present in our macrophage cultures were functionally signficant for our key endpoints. Macrophages treated with a blocking antibody against IFN-γ (Figures S9A and S9B) and *Ifng*$^{-/-}$ macrophages (Figures S9C and S9D) showed reduced maturation of *Mtb* containing phagosomes. In the case of *Ifng*$^{-/-}$ macrophages, maturation was restored by adding exogenous IFN-γ (Figure S9D). *Ifng*$^{-/-}$ macrophages also showed reduced autophagy in response to *Mtb* (Figures S9E and S9F). Finally, *Ifng*$^{-/-}$ macrophages showed a defect in bactericidal activity as assessed by intracellular bacterial burden at 72 hr, similar to that seen in *Tirap*$^{-/-}$ macrophages (Figure S9G). These data demonstrated that macrophages secrete small but functionally relevant amounts of IFN-γ.

The S200L/S180L Mal Mutation Reduces IFN-γ Signaling

Given the new role identified for Mal in IFN-γ signaling, we then proceeded to look at the effect of the S200L polymorphism on TLR2, TLR4, and IFN-γ signaling in macrophages. As noted above, the S200L mutation did not affect secretion of the pro-inflammatory cytokine TNF-α in response to the TLR2 ligands Malp-2 and Pam_3CysK_4 or the TLR4 ligand LPS (Figures S2A–S2C) or *Tnfa* mRNA levels in response to TLR2 and TLR4 ligands (Figure 6B). However, macrophages from LL mice and, to a lesser extent, SL mice, showed a decreased IP-10 response to IFN-γ stimulation (Figures 6A and 6C), indicating that carriage of the 200L allele in mice, impairs IFN-γ, but not TLR, responses.

We then sought to determine the effect of the S180L mutation in human macrophages. We derived macrophages from peripheral blood monocytes (MDMs) from donors genotyped using allelic discrimination. MDMs were stimulated with TLR ligands and TNF-α secretion was measured by ELISA. We observed decreased responses to TLR2 and TLR4 ligands in human MDMs (Figure 6E). We observed a decrease in IFN-γ-driven IP-10 secretion in MDMs from individuals who were homozygous for the S180L mutation (Figure 6D). We proceeded to assess whether the S180L mutation altered the affinity of Mal for the IFNGR. Using site-directed mutagenesis, we synthesized HA-tagged human Mal with the S180L mutation (180L). S180L variant human Mal shows decreased affinity for the IFNGR relative to HA-tagged wild-type Mal (Figure 6F). The S180L mutation reduces the affinity of Mal for the IFNGR and reduces IP-10 secretion in response to IFN- γ stimulation.

DISCUSSION

Here we show that the murine equivalent of the S180L mutation in Mal, Mal S200L, replicated the phenotype previously reported from humans with the S180L polymorphism: it conferred protection from tuberculous disease on heterozygotes for the mutation and increased susceptibility on homozygotes. In vitro, Mal S200L impaired phagosome maturation and killing of intracellular mycobacteria. However, unlike S180L, S200L did not affect TLR signaling. We demonstrated a mechanism for these observations: the S200L polymorphism affected a TLR-independent, Mal-dependent, IFNGR signaling pathway. Mal-dependent IFNGR signaling was required for p38 phosphorylation, autophagy, phagosome maturation, and killing of intracellular mycobacteria. This IFNGR signaling pathway was attenuated by the human mutation S180L. This offers an explanation for how the common S180L mutation affects host innate immune responses to *Mtb.* The fact that this common polymorphism attenuates IFNGR signaling might have relevance for host susceptibility to a number of IFN-γ-related conditions including autoimmunity and cancers.

A number of publications regarding the effect of the S180L SNP on infectious disease susceptibility in humans have reported a heterozygote advantage with increased susceptibility seen in homozygotes (Capparelli et al., 2013; Castiblanco et al., 2008; Khor et al., 2007; Selvaraj et al., 2010). A murine model using the equivalent of the S180L mutation, S200L,

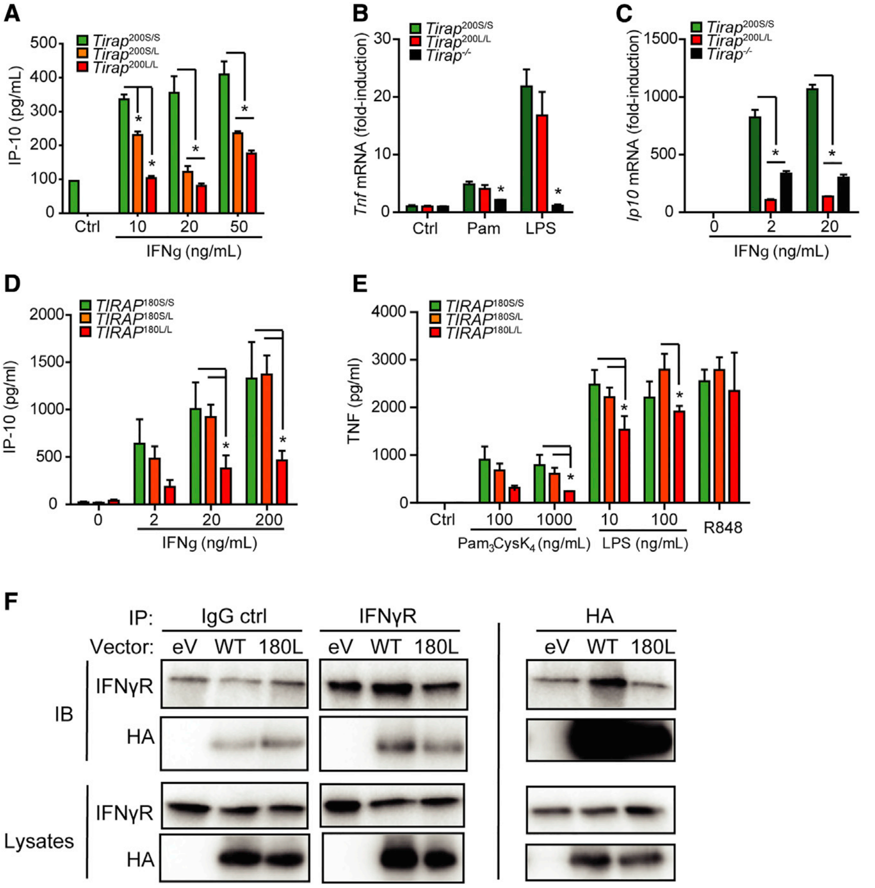

Figure 6. The S180L/S200L Mutation Reduces Affinity for IFNγR1 and Impairs Responses to IFN-γ

(A) IP-10 secretion by primary murine BMM stimulated for 20 hr with rmIFN-γ and supernatants analyzed by ELISA, mean ± SD pooled from three separate experiments with macrophages from one mouse per group in each experiment.

(B and C) *Tnfa* and *Ip-10* mRNA levels from iBMM stimulated for 4 hr with Pam_3Cys_4K (100 μg/mL), LPS (100 ng/ml) or rmIFN-γ at the concentrations indicated.

(D and E) Monocyte-derived macrophages from human volunteers genotyped for the S180L SNP (n = 12 for *TIRAP* 180S/S, n = 12 for *TIRAP* 180S/L, and n = 4 for *TIRAP* 180L/L) were stimulated with rhIFN-γ (D) or TLR ligands (E) for 16 hr. Supernatants were analyzed for IP-10 production (D) or TNF (E) by ELISA. Mean ± SD shown in graph, analyzed using two-way ANOVA.

(F) HEK293 cells were transfected with HA-tagged wild-type, HA-tagged S180L variant Mal, or HA-tagged empty vector and an immunoprecipitation was performed with antibodies to HA and IFNGR1 on cell lysates as indicated prior to Western blotting.

replicated these findings, with heterozygotes protected from weight loss (a cardinal clinical feature of human tuberculosis) and homozygotes displaying increased bacterial burden and lung inflammation.

In vitro, macrophages from mice carrying the L allele displayed impaired phagosome maturation and killing of *Mtb* but did not show evidence of impaired cytokine production. In contrast, macrophages from TLR-deficient mice displayed impaired cytokine production but preserved phagosome maturation and killing of *Mtb.* Mal-deficient macrophages displayed impairment of cytokine production in addition to the phenotype seen in the S180L macrophages of impaired phagosome maturation and killing.

These findings led us to search for a TLR-independent function for Mal. We show here a TLR2- and TLR4-independent role for Mal in the IFNGR signaling pathway. Mal bound to the IFNGR and MyD88 and acted as a bridging adaptor between these proteins. Mal was required for IP-10 production in response to IFN-γ and for a pathway involving p38 MAPK phosphorylation, culminating in autophagy and killing of intracellular bacteria. Autophagy is a key means of killing intracellular bacteria, including *Mtb*, and also plays a role in regulation of cytokine secretion (Ní Cheallaigh et al., 2011; Peral de Castro et al., 2012). IFN-γ-induced phagosome maturation is dependent on Beclin-1 and might indeed be autophagy-dependent (Harris et al., 2007). The defect in autophagy seen in the absence of Mal-dependent IFNGR signaling provides an explanation for why Mal and MyD88, but not TLRs 2 and 4, are required for macrophage killing of *Mtb.*

IFN-γ is a canonical macrophage activator and is known to play a critical role in immune responses to *Mtb* (Bogunovic et al., 2012; Cooper et al., 1993; Filipe-Santos et al., 2006; Flesch and Kaufmann, 1991; Flynn et al., 1993). IFN-γ is secreted in large quantities by activated Th1 cells (Mosmann and Coffman, 1989), activated $CD8^+$ cytotoxic cells (Sad et al., 1995), and NK cells (Perussia, 1991). However, our model for bactericidal activity and autophagy consisted of a monoculture of macrophages. There are numerous reports of macrophages secreting IFN-γ (Darwich et al., 2009; Di Marzio et al., 1994; Fenton et al., 1997; Fultz et al., 1993), albeit in limited quantities, although some authors have highlighted the possible effect of contaminating cells in producing IFN-γ (Schleicher et al., 2005). The increased proportion of IFN-γ producing cells in macrophages pre-stimulated with LPS and then "recovered" (O'Carroll et al., 2014) provides compelling evidence that macrophages can produce IFN-γ given appropriate stimuli.

We show here that the murine immortalized and primary macrophages used in our *Mtb* infection model secreted small but functionally decisive quantities of IFN-γ in response to infection with *Mtb* and that Mal played a critical role in macrophage responses to *Mtb*, which are IFN-γ dependent.

Given our identification of a role for Mal in IFNGR signaling and its established role in TLR2 and TLR4 signaling, we examined the effect of the S180L polymorphism and the murine equivalent of the S180L polymorphism, S200L, on these pathways. We show that responses to IFN-γ are reduced in human cells with the 180L variant. Human 180L variant Mal showed a reduced affinity for the IFNGR. Cells from mice with the equivalent mutation also showed impairment in IFNGR signaling.

Human S180L reduced responses to TLR2 and TLR4 ligands, consistent with the known role of Mal as a signaling adaptor protein downstream of TLRs 2 and 4, and with published data on the role of TLR2 as a pattern-recognition receptor involved in pro-inflammatory cytokine production by *Mtb*-infected macrophages. Previously published data have shown that Mal is not required for responses to high doses of TLR2 ligands (Kenny et al., 2009); however, we hypothesize that the doses of *Mtb* used in this study correspond to the lower doses of TLR2 ligands for which Mal is required. Our findings that S180L reduced responses to TLR 2 and 4 ligands are consistent with a previous report using L-variant Mal transfected into murine MEFs (Khor et al., 2007) and with a recent study where PBMCs from individuals with the Mal S180L allele were stimulated with heat-killed *Mtb* (Capparelli et al., 2013). In contrast, another publication reported no difference between TLR2 and TLR4 signaling in vitro in PBMCs from individuals with the SS and SL genotypes but did show an increase in response to low doses of TLR2 ligand in PBMCs from a single individual with the LL genotype (Ferwerda et al., 2009). This finding might have been caused by the presence of polymorphisms in MyD88 which interact with the Mal SNP (Capparelli et al., 2013).

Macrophages from mice homozygous for the S200L mutation, the murine equivalent of S180L, did not show an impairment in TLR signaling but did show an impairment in IFN-γ signaling. Murine Mal differs from human Mal in that it has 20 more amino acids at the N terminus. Mutations in Mal might selectively impair certain signaling pathways only, as demonstrated by a recent report on a form of Mal with an altered lipid binding domain, which selectively impaired responses to the TLR9 ligand CpG but not the TLR4 ligand LPS (Bonham et al., 2014).

Importantly, the selective impairment of IFN-γ but not TLR signaling in S200L macrophages meant that the reduced phagosomal maturation and bacterial killing in macrophages with the L allele and the increased bacterial burden and increased inflammatory response seen in homozygote S200L mice could be the result of aberrant IFN-γ signaling associated with the S180L SNP rather than the result of impairment in TLR signaling. The increased inflammation seen in the homozygote mutant mice might reflect the increase in bacterial burden in the homozygote mice or might reflect a loss of IFN-γ-mediated regulation of neutrophil recruitment and inflammation (Mishra et al., 2013; Nandi and Behar, 2011). The increase in bacterial burden and inflammation is not as marked as the defect seen in mice entirely deficient in IFN-γ (Cooper et al., 1993; Flynn et al., 1993), but could be consistent with a partial defect in IFN-γ signaling.

Macrophage in vitro assays and assessment of bacterial burden and inflammation following infection demonstrated a disadvantage for homozygote mutants, whereas serial weights demonstrated increased severity of disease in both WT and mutant homozygote individuals. A number of publications on the S180L SNP and TB susceptibility in humans have reported a heterozygote advantage and/or increased susceptibility in mutant homozygotes. A heterozygote advantage in vivo has also been reported for a SNP in leukotriene A4 hydrolase (LTA4), with impaired in vitro responses seen in homozygotes for the mutation. This effect has been attributed to the effect of the SNP on mitochondrial reactive oxygen species production and cell necrosis (Roca and Ramakrishnan, 2013). As we face an era of drug-resistant tuberculosis, improved understanding of common mutations, which affect inflammatory responses to pathogens such as Mal S180L, might guide us to improved use of immunomodulatory treatment. Tobin and colleagues have highlighted the potential of using LTA4H host genotypes to guide choice of treatment for tuberculosis: individuals with tuberculous meningitis with a LTA4 genotype associated with an excessive inflammatory response benefited from adjunctive immunomodulatory steroid treatment, whereas this was harmful in those with a LTA4 genotype associated with an inadequate inflammatory response (Tobin et al., 2012). It is tempting to speculate that the Mal S180L genotype could be used to inform treatment decisions in a similar manner.

The S180L SNP in Mal has been reported to be associated with altered susceptibility to not only mycobacterial disease, but also to other infectious diseases including pneumococcal disease, malaria, and Chaga's disease. IFN-γ signaling also plays an important role in immune responses to these pathogens. Hitherto, the association of systemic lupus erythematosus (SLE) with S180L has been unexplained. TLR2 and TLR4 are not thought to play an important role in SLE (Kim et al., 2009), although TLR 9 has been implicated (Yang et al., 2012). Our data suggest that the increased susceptibility to SLE associated with S180L might be due to defects in the Mal-dependent IFN-γ signaling pathway rather than alterations in TLR2 and 4 signaling (Pollard et al., 2013).

Indeed, IFN-γ has a critical role in immunity, including promoting differentiation of macrophages to a classically activated phenotype (Martinez and Gordon, 2014). IFN-γ plays a role across a spectrum of non-infectious diseases including atherogenesis, autoimmune diseases such as chronic atopic dermatitis, and cancer (Feingold, 2014; Ikeda et al., 2002; Schroecksnadel et al., 2006). Given the frequency of the Mal S180L SNP, it might be fruitful to assess whether the Mal-dependent IFN-γ signaling pathway plays a role in these diseases.

EXPERIMENTAL PROCEDURES

Cell Lines and Culture

Primary bone-marrow-derived macrophages were derived from the femurs of *Tirap*$^{-/-}$, *Ifng*$^{-/-}$, and WT mice and differentiated for 7 days with medium containing MCSF. THP-1 cells (ATCC) were transfected with siRNA against Mal (Dharmacon) and scrambled control prior to being differentiated into macrophage-like cells with phorbol myristate acetate (100 nmol/L).

Assessment of Bacterial Growth

Macrophages were grown at 1×10^5 cells/ml in 12-well plates in antibiotic-free RPMI supplemented with 10% fetal calf serum. A suspension of *Mtb* H37Rv was prepared as described in Supplemental Experimental Procedures, and macrophages were infected with *Mtb* at a MOI of 10 bacteria/macrophage. Extracellular bacteria were washed off at 3 hr post-infection. Cells were lysed at the indicated time points and bacteria were harvested and colonies were counted approximately 21 days later.

Phagosome Maturation Assays

Mtb H37Rv was labeled with FITC (1 mg/ml, Sigma). Bacteria were incubated with cells for 1 hr prior to the addition of LysoTracker Red DND-99 (Invitrogen) (100 nmol/L) for 1 hr prior to fixation. Alternatively, cells were fixed, permeabilized, and with anti-CD63 /LAMP-3 (Santa Cruz Biotechnology) (1 μg/mL) followed by secondary antibody. Images were recorded on an Olympus FluoView 1000 and a Zeiss LSM 510 laser scanning confocal microscope.

Autophagy Analysis

Autophagosome formation was measured by LC3 punctate staining using LC3 antibody (Invitrogen). Autophagic flux was inhibited using either bafilomycin (100mM) or a combination of E64d and pepstatin.

Cytokine Measurements

Cytokine measurements were performed in supernatants using commercial ELISA kits.

Co-Immunoprecipitation

HEK293 cells were incubated for 24 hr with DNA encoding various proteins in the presence of Genejuice. Immunoprecipitation was initiated by incubation of lysates for 2 hr with protein A/G sepharose beads (Amersham) plus control antibodies. Precleared lysates were then incubated at 4°C for at least 2 hr with various antibodies and protein G beads (Amersham) prior to separation by SDS- PAGE and visualization with an enhanced chemiluminescence system (Li-Cor).

Plasmids

HA-Mal has been previously described (Valkov et al., 2011). Site-directed mutagensis was carried out to generate HA-tagged S180L variant Mal which was amplified using Miniprep (QIAGEN). The sequences of both HA-Mal and HA-S180L Mal were confirmed by seqencing (Eurofins).

Fluorescence Lifetime Imaging Microscopy-Fluorescence Resonance Energy Transfer

Macrophages were stained with primary antibodies against IFNGR2 (MyBioSource) and MyD88 (Abcam) and secondary antibodies Alexa Fluor A488 (donor) and A568 (acceptor) antibodies. An Olympus FV1000 microscope equipped with a PicoHarp300 FLIM extension and a 485 nm pulsed laser diode from PicoQuant was used to record FLIM data.

Genotyping

Mal genotype was determined on DNA extracted from buccal swabs (Isohelix, Cell Product). Genotyping of the Mal S180L and polymorphism was performed using the TaqMan Allelic Discrimination System (PE Biosystems).

FACS Analysis

Immortalized macrophages were stimulated, fixed, and stained with anti-pSTAT1 (Y701) antibody conjugated to AlexaFluor 488 before analysis on a BD FACSCanto II analyzer. For analysis of intracellular IFN-γ production, macrophages were infected Mtb H37Rv and then incubated with Brefeldin A. Cells were permeabilized and stained with an anti-mouse IFN-γ antibody (BD) or isotype control.

Mice

Mal S200L heterozygote and homozygote mice (C57BL/6 background) were generated as described in Figure S1. S200L mice were generated with C57BL/6 embryonic stem cells and C57BL/6 blastocysts. Mice were age and sex matched. Mice were infected with Mtb via aerosol (Martens et al., 2012) at approximately 8 weeks of age.

Bacterial Load

At 8 weeks, mice were sacrificed. Lung homogenates from six mice were plated to measure bacterial burden.

Lung Histology

Lungs were inflated, fixed, and stained (H&E). Lung surface area of inflammation was measured with a Nikon Eclipse E400. Percent total lung area involved with inflammation was calculated by dividing the cumulative area of inflammation by the total lung surface area examined for each lung studied.

Lung Cytokine Expression

Lung lysates were assayed for TNF-α by ELISA (R&D Systems).

Statistical Analysis

A one-way ANOVA was performed to assess for statistically significant difference of the means between groups. Chi-square analysis was used to assess statistically significant proportions of co-localization between groups. p values < 0.05 were considered significant. Error bars represent SD of the mean.

SUPPLEMENTAL INFORMATION

Supplemental Information includes six figures and Supplemental Experimental Procedures and can be found with this article online at http://dx.doi.org/10.1016/j.immuni.2016.01.019.

AUTHOR CONTRIBUTIONS

Conceptualization: C.N.C., F.J.S., J.H., N.M.-W., S.V.G., L.A.J.O.N., E.C.L., and J.K.; Methodology: C.N.C., F.J.S., J.H., J.L., N.M.-W., E.P.M., M.M.H., S.A.J., and S.V.G.; Investigation: C.N.C., F.J.S., J.H., N.M.-W., J.L., K.W., E.P.M., A.S., L.E.G., M.C., N.M., C.H.A.H., G.A.T., E.C.C., S.A.J., M.M.H., S.C.C., M.O.S., and C.M.F.; Formal Analysis: C.N.C., F.J.S., J.H., N.M.-W., and S.A.J.; Resources: S.C.C., N.J.B., D.G., H.K., S.V.G., and L.A.J.O.N.; Writing – Original Draft: C.N.C.; Writing – Review & Editing: C.N.C., F.J.S., J.H., H.K., E.C.L., and J.K.; Funding Acquisition: C.N.C., L.A.J.O.N., E.C.L., and J.K.; Supervision: H.K., S.V.G., L.A.J.O.N., E.C.L., and J.K. F.J.S. and J.H. contributed equally to this work.

ACKNOWLEDGMENTS

Supported in part by Health Research Board Ireland by grant NSAFP 2009/2 (C.N.C.), CSA/2004/7 and CSA/2012/16 (J.K.), The Royal City of Dublin Hospital Trust (J.K.), NIH grant HL081149 (H.K.), Wellcome Trust PhD award 102395/Z/13/Z (A.S.), Science Foundation Ireland Investigator Awards 08/IN.1/B2038 (S.G.) and 12/IA/1421 (E.C.L.), and the Science Foundation Ireland Strategic Research Cluster 07/SRC/B1144 (E.C.L.).

Received: August 26, 2014
Revised: April 20, 2015
Accepted: November 6, 2015
Published: February 16, 2016

REFERENCES

Bogunovic, D., Byun, M., Durfee, L.A., Abhyankar, A., Sanal, O., Mansouri, D., Salem, S., Radovanovic, I., Grant, A.V., Adimi, P., et al. (2012). Mycobacterial disease and impaired IFN-γ immunity in humans with inherited ISG15 deficiency. Science *337*, 1684–1688.

Bonham, K.S., Orzalli, M.H., Hayashi, K., Wolf, A.I., Glanemann, C., Weninger, W., Iwasaki, A., Knipe, D.M., and Kagan, J.C. (2014). A promiscuous lipid-binding protein diversifies the subcellular sites of toll-like receptor signal transduction. Cell *156*, 705–716.

Capparelli, R., De Chiara, F., Di Matteo, A., Medaglia, C., and Iannelli, D. (2013). The MyD88 rs6853 and TIRAP rs8177374 polymorphic sites are associated with resistance to human pulmonary tuberculosis. Genes Immun. *14*, 504–511.

Castiblanco, J., Varela, D.-C., Castaño-Rodríguez, N., Rojas-Villarraga, A., Hincapié, M.-E., and Anaya, J.-M. (2008). TIRAP (MAL) S180L polymorphism is a common protective factor against developing tuberculosis and systemic lupus erythematosus. Infect. Genet. Evol. *8*, 541–544.

Cooper, A.M., Dalton, D.K., Stewart, T.A., Griffin, J.P., Russell, D.G., and Orme, I.M. (1993). Disseminated tuberculosis in interferon gamma gene-disrupted mice. J. Exp. Med. *178*, 2243–2247.

Darwich, L., Coma, G., Peña, R., Bellido, R., Blanco, E.J., Este, J.A., Borras, F.E., Clotet, B., Ruiz, L., Rosell, A., et al. (2009). Secretion of interferon-gamma by human macrophages demonstrated at the single-cell level after costimulation with interleukin (IL)-12 plus IL-18. Immunology *126*, 386–393.

Deretic, V., Singh, S., Master, S., Harris, J., Roberts, E., Kyei, G., Davis, A., de Haro, S., Naylor, J., Lee, H.H., and Vergne, I. (2006). Mycobacterium tuberculosis inhibition of phagolysosome biogenesis and autophagy as a host defence mechanism. Cell. Microbiol. *8*, 719–727.

Di Marzio, P., Puddu, P., Conti, L., Belardelli, F., and Gessani, S. (1994). Interferon gamma upregulates its own gene expression in mouse peritoneal macrophages. J. Exp. Med. *179*, 1731–1736.

Dissanayeke, S.R., Levin, S., Pienaar, S., Wood, K., Eley, B., Beatty, D., Henderson, H., Anderson, S., and Levin, M. (2009). Polymorphic variation in TIRAP is not associated with susceptibility to childhood TB but may determine susceptibility to TBM in some ethnic groups. PLoS ONE *4*, e6698.

Feingold, K.R. (2014). The adverse effect of IFN gamma on stratum corneum structure and function in psoriasis and atopic dermatitis. J. Invest. Dermatol. *134*, 597–600.

Fenton, M.J., Vermeulen, M.W., Kim, S., Burdick, M., Strieter, R.M., and Kornfeld, H. (1997). Induction of gamma interferon production in human alveolar macrophages by Mycobacterium tuberculosis. Infect. Immun. *65*, 5149–5156.

Ferwerda, B., Alonso, S., Banahan, K., McCall, M.B., Giamarellos-Bourboulis, E.J., Ramakers, B.P., Mouktaroudi, M., Fain, P.R., Izagirre, N., Syafruddin, D., et al. (2009). Functional and genetic evidence that the Mal/TIRAP allele variant 180L has been selected by providing protection against septic shock. Proc. Natl. Acad. Sci. USA *106*, 10272–10277.

Filipe-Santos, O., Bustamante, J., Chapgier, A., Vogt, G., de Beaucoudrey, L., Feinberg, J., Jouanguy, E., Boisson-Dupuis, S., Fieschi, C., Picard, C., and Casanova, J.L. (2006). Inborn errors of IL-12/23- and IFN-gamma-mediated immunity: molecular, cellular, and clinical features. Semin. Immunol. *18*, 347–361.

Fitzgerald, K.A., Palsson-McDermott, E.M., Bowie, A.G., Jefferies, C.A., Mansell, A.S., Brady, G., Brint, E., Dunne, A., Gray, P., Harte, M.T., et al. (2001). Mal (MyD88-adapter-like) is required for Toll-like receptor-4 signal transduction. Nature *413*, 78–83.

Flesch, I.E., and Kaufmann, S.H. (1991). Mechanisms involved in mycobacterial growth inhibition by gamma interferon-activated bone marrow macrophages: role of reactive nitrogen intermediates. Infect. Immun. *59*, 3213–3218.

Flynn, J.L., Chan, J., Triebold, K.J., Dalton, D.K., Stewart, T.A., and Bloom, B.R. (1993). An essential role for interferon gamma in resistance to Mycobacterium tuberculosis infection. J. Exp. Med. *178*, 2249–2254.

Fultz, M.J., Barber, S.A., Dieffenbach, C.W., and Vogel, S.N. (1993). Induction of IFN-gamma in macrophages by lipopolysaccharide. Int. Immunol. *5*, 1383–1392.

Gutierrez, M.G., Master, S.S., Singh, S.B., Taylor, G.A., Colombo, M.I., and Deretic, V. (2004). Autophagy is a defense mechanism inhibiting BCG and Mycobacterium tuberculosis survival in infected macrophages. Cell *119*, 753–766.

Harris, J., De Haro, S.A., Master, S.S., Keane, J., Roberts, E.A., Delgado, M., and Deretic, V. (2007). T helper 2 cytokines inhibit autophagic control of intracellular Mycobacterium tuberculosis. Immunity *27*, 505–517.

Harris, J., Hope, J.C., and Lavelle, E.C. (2009). Autophagy and the immune response to TB. Transbound. Emerg. Dis. *56*, 248–254.

Hölscher, C., Reiling, N., Schaible, U.E., Hölscher, A., Bathmann, C., Korbel, D., Lenz, I., Sonntag, T., Kröger, S., Akira, S., et al. (2008). Containment of aerogenic Mycobacterium tuberculosis infection in mice does not require MyD88 adaptor function for TLR2, -4 and -9. Eur. J. Immunol. *38*, 680–694.

Horng, T., Barton, G.M., and Medzhitov, R. (2001). TIRAP: an adapter molecule in the Toll signaling pathway. Nat. Immunol. *2*, 835–841.

Horng, T., Barton, G.M., Flavell, R.A., and Medzhitov, R. (2002). The adaptor molecule TIRAP provides signalling specificity for Toll-like receptors. Nature *420*, 329–333.

Ikeda, H., Old, L.J., and Schreiber, R.D. (2002). The roles of IFN γ in protection against tumor development and cancer immunoediting. Cytokine Growth Factor Rev. *13*, 95–109.

Kagan, J.C., and Medzhitov, R. (2006). Phosphoinositide-mediated adaptor recruitment controls Toll-like receptor signaling. Cell *125*, 943–955.

Kenny, E.F., Talbot, S., Gong, M., Golenbock, D.T., Bryant, C.E., and O'Neill, L.A. (2009). MyD88 adaptor-like is not essential for TLR2 signaling and inhibits signaling by TLR3. J. Immunol. *183*, 3642–3651.

Khor, C.C., Chapman, S.J., Vannberg, F.O., Dunne, A., Murphy, C., Ling, E.Y., Frodsham, A.J., Walley, A.J., Kyrieleis, O., Khan, A., et al. (2007). A Mal functional variant is associated with protection against invasive pneumococcal disease, bacteremia, malaria and tuberculosis. Nat. Genet. *39*, 523–528.

Kim, W.U., Sreih, A., and Bucala, R. (2009). Toll-like receptors in systemic lupus erythematosus; prospects for therapeutic intervention. Autoimmun. Rev. *8*, 204–208.

Ladhani, S.N., Davila, S., Hibberd, M.L., Heath, P.T., Ramsay, M.E., Slack, M.P., Pollard, A.J., and Booy, R. (2010). Association between single-nucleotide polymorphisms in Mal/TIRAP and interleukin-10 genes and susceptibility to invasive haemophilus influenzae serotype b infection in immunized children. Clin. Infect. Dis. *51*, 761–767.

Liu, Q., Li, W., Li, D., Feng, Y., and Tao, C. (2014). TIRAP C539T Polymorphism Contributes to Tuberculosis Susceptibility: Evidence from a Meta-Analysis. Infect Genet Evol.

MacMicking, J.D. (2012). Interferon-inducible effector mechanisms in cell-autonomous immunity. Nat. Rev. Immunol. *12*, 367–382.

Martens, G.W., Vallerskog, T., and Kornfeld, H. (2012). Hypercholesterolemic LDL receptor-deficient mice mount a neutrophilic response to tuberculosis despite the timely expression of protective immunity. J. Leukoc. Biol. *91*, 849–857.

Martinez, F.O., and Gordon, S. (2014). The M1 and M2 paradigm of macrophage activation: time for reassessment. F1000Prime Rep. *6*, 13.

Matsuzawa, T., Fujiwara, E., and Washi, Y. (2014). Autophagy activation by interferon-γ via the p38 mitogen-activated protein kinase signalling pathway is involved in macrophage bactericidal activity. Immunology *141*, 61–69.

Mishra, B.B., Rathinam, V.A., Martens, G.W., Martinot, A.J., Kornfeld, H., Fitzgerald, K.A., and Sassetti, C.M. (2013). Nitric oxide controls the immunopathology of tuberculosis by inhibiting NLRP3 inflammasome-dependent processing of IL-1β. Nat. Immunol. *14*, 52–60.

Mosmann, T.R., and Coffman, R.L. (1989). TH1 and TH2 cells: different patterns of lymphokine secretion lead to different functional properties. Annu. Rev. Immunol. *7*, 145–173.

Nandi, B., and Behar, S.M. (2011). Regulation of neutrophils by interferon-γ limits lung inflammation during tuberculosis infection. J. Exp. Med. *208*, 2251–2262.

Nejentsev, S., Thye, T., Szeszko, J.S., Stevens, H., Balabanova, Y., Chinbuah, A.M., Hibberd, M., van de Vosse, E., Alisjahbana, B., van Crevel, R., et al. (2008). Analysis of association of the TIRAP (MAL) S180L variant and tuberculosis in three populations. Nat. Genet. *40*, 261–262, author reply 262–263.

Ní Cheallaigh, C., Keane, J., Lavelle, E.C., Hope, J.C., and Harris, J. (2011). Autophagy in the immune response to tuberculosis: clinical perspectives. Clin. Exp. Immunol. *164*, 291–300.

O'Carroll, C., Fagan, A., Shanahan, F., and Carmody, R.J. (2014). Identification of a unique hybrid macrophage-polarization state following recovery from lipopolysaccharide tolerance. J. Immunol. *192*, 427–436.

Peral de Castro, C., Jones, S.A., Ní Cheallaigh, C., Hearnden, C.A., Williams, L., Winter, J., Lavelle, E.C., Mills, K.H.G., and Harris, J. (2012). Autophagy regulates IL-23 secretion and innate T cell responses through effects on IL-1 secretion. J. Immunol. *189*, 4144–4153.

Perussia, B. (1991). Lymphokine-activated killer cells, natural killer cells and cytokines. Curr. Opin. Immunol. *3*, 49–55.

Pollard, K.M., Cauvi, D.M., Toomey, C.B., Morris, K.V., and Kono, D.H. (2013). Interferon-γ and systemic autoimmunity. Discov. Med. *16*, 123–131.

Roca, F.J., and Ramakrishnan, L. (2013). TNF dually mediates resistance and susceptibility to mycobacteria via mitochondrial reactive oxygen species. Cell *153*, 521–534.

Sad, S., Marcotte, R., and Mosmann, T.R. (1995). Cytokine-induced differentiation of precursor mouse CD8+ T cells into cytotoxic CD8+ T cells secreting Th1 or Th2 cytokines. Immunity *2*, 271–279.

Schleicher, U., Hesse, A., and Bogdan, C. (2005). Minute numbers of contaminant CD8+ T cells or CD11b+CD11c+ NK cells are the source of IFN-gamma in

IL-12/IL-18-stimulated mouse macrophage populations. Blood *105*, 1319–1328.

Schroecksnadel, K., Frick, B., Winkler, C., and Fuchs, D. (2006). Crucial role of interferon-gamma and stimulated macrophages in cardiovascular disease. Curr. Vasc. Pharmacol. *4*, 205–213.

Selvaraj, P., Harishankar, M., Singh, B., Jawahar, M.S., and Banurekha, V.V. (2010). Toll-like receptor and TIRAP gene polymorphisms in pulmonary tuberculosis patients of South India. Tuberculosis (Edinb.) *90*, 306–310.

Sun, D., and Ding, A. (2006). MyD88-mediated stabilization of interferon-gamma-induced cytokine and chemokine mRNA. Nat. Immunol. *7*, 375–381.

Tobin, D.M., Roca, F.J., Oh, S.F., McFarland, R., Vickery, T.W., Ray, J.P., Ko, D.C., Zou, Y., Bang, N.D., Chau, T.T., et al. (2012). Host genotype-specific therapies can optimize the inflammatory response to mycobacterial infections. Cell *148*, 434–446.

Valkov, E., Stamp, A., Dimaio, F., Baker, D., Verstak, B., Roversi, P., Kellie, S., Sweet, M.J., Mansell, A., Gay, N.J., et al. (2011). Crystal structure of Toll-like receptor adaptor MAL/TIRAP reveals the molecular basis for signal transduction and disease protection. Proc. Natl. Acad. Sci. USA *108*, 14879–14884.

Watson, R.O., Manzanillo, P.S., and Cox, J.S. (2012). Extracellular M. tuberculosis DNA targets bacteria for autophagy by activating the host DNA-sensing pathway. Cell *150*, 803–815.

Yamamoto, A., Tagawa, Y., Yoshimori, T., Moriyama, Y., Masaki, R., and Tashiro, Y. (1998). Bafilomycin A1 prevents maturation of autophagic vacuoles by inhibiting fusion between autophagosomes and lysosomes in rat hepatoma cell line, H-4-II-E cells. Cell Struct. Funct. *23*, 33–42.

Yamamoto, M., Sato, S., Hemmi, H., Sanjo, H., Uematsu, S., Kaisho, T., Hoshino, K., Takeuchi, O., Kobayashi, M., Fujita, T., et al. (2002). Essential role for TIRAP in activation of the signalling cascade shared by TLR2 and TLR4. Nature *420*, 324–329.

Yang, Z., Liang, Y., Qin, B., Li, C., and Zhong, R. (2012). TLR9 polymorphisms and systemic lupus erythematosus risk in Asians: a meta-analysis study. Cytokine *57*, 282–289.

Immunity, Volume *44*

Supplemental Information

A Common Variant in the Adaptor Mal Regulates Interferon Gamma Signaling

Clíona Ní Cheallaigh, Frederick J. Sheedy, James Harris, Natalia Muñoz-Wolf, Jinhee Lee, Kim West, Eva Palsson McDermott, Alicia Smyth, Laura E. Gleeson, Michelle Coleman, Nuria Martinez, Claire H.A. Hearnden, Graham A. Tynan, Elizabeth C. Carroll, Sarah A. Jones, Sinéad C. Corr, Nicholas J. Bernard, Mark M. Hughes, Sarah E. Corcoran, Mary O'Sullivan, Ciara M. Fallon, Hardy Kornfeld, Douglas Golenbock, Stephen V. Gordon, Luke A.J. O'Neill, Ed C. Lavelle, and Joseph Keane

A.

```
human Mal    MASSTSLPAPGSRPKKPLGKMADWFRQTLLKKPKKRPNSPESTSSDASQP 50
murine Mal   MASSSSVPASSTPSKKPRDKIADWFRQALLKKPKKMPISQESHLYDGSQT 50
             ****:*:**..: .*** .*:******:******* * * **   *.**.

human Mal    TSQD--SP----------------LPPSLSSVTSPSLPPTHASDSGSS-- 80
murine Mal   ATQDGLSPSSCSSPPSHSSPESRSSPSSCSSGMSPTSPPTHVDSSSSSSG 100
             ::**  **                *.* **  **: ****...*.**

human Mal    RWSKDYDVCVCHSEEDLVAAQDLVSYLEGSTASLRCFLQLRDATPGGAIV 130
murine Mal   RWSKDYDVCVCHSEEDLEAAQELVSYLEGSQASLRCFLQLRDAAPGGAIV 150
             ***************** ***:******** ************:******

human Mal    SELCQALSSSHCRVLLITPGFLQDPWCKYQMLQALTEAPGAEGCTIPLLS 180
murine Mal   SELCQALSRSHCRALLITPGFLRDPWCKYQMLQALTEAPASEGCTIPLLS 200
             ******** ****.********:****************.:*********

human Mal    GLSRAAYPPELRFMYYVDGRGPDGGFRQVKEAVMRYLQTLSWHLLYHGTP 230
murine Mal   GLSRAAYPPELRFMYYVDGRGKDGGFYQVKEAVIHYLETLS--------- 241
             ********************* **** ******::**:***

human Mal    EIGVKLETENPCRASDSHKCDKRYRE 256
             --------------------------
```

B.

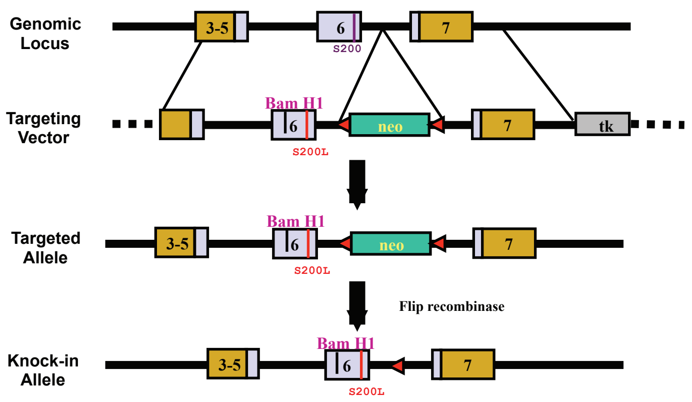

C.

Tirap-180:	human	mouse
WT allele	TCG=(Ser)	TCC=(Ser)
mutant allele	TTG=(Leu)	CTC=(Leu)

D.

E6

```
ACTGGTTCAGGCAGGCTCTGTTGAAGAAGCCCAAGAAGATGCCGATCTCCCAGGAAAGCC
ACCTCTATGATGGTTCACAGACAGCCACACAGGATGGTCTCTCACCCTCGAGCTGCAGCT
CACCCCCGAGTCACAGTTCACCGGAGAGCCGTAGCTCACCCTCGAGCTGCAGTTCAGGAA
TGTCACCTACCTCGCCACCAACACACGTGGACAGCAGCAGCAGCAGCAGTGGCCGCTGGA
GCAAAGACTACGATGTCTGCGTGTGCCACAGTGAGGAGGACTTGGAGGCGGCCCAGGAGC
TGGTCTCCTACTTGGAGGGatcCCAGGCCAGTCTACGCTGCTTCCTGCAGCTTCGGGATG
CAGCCCCGGGTGGCGCCATTGTTTCGGAGCTATGCCAGGCACTGAGTCGTAGTCACTGCC
GTGTGCTGCTCATCACTCCAGGCTTCCTTCGGGACCCCTGGTGCAAGTACCAGATGCTGC
AGGCCCTGACGGAGGCCCCGGCGTCGGAGGGTTGCACCATACCCCTGCTGctCGGCCTGT
CCAGAGCCGCCTATCCGCCGGAACTCCGATTCATGTACTATGTGGATGGCAGAGGCAAGG
ACGGAGGCTTTTACCAAGTCAAGGAGGCTGTTATACACT
```

Supplementary Figure 1 - Design and generation of mutant *Tirap* S200L mice, the equivalent mutation of human S180L. A) Clustal alignment of human and murine Mal protein sequences. Serine 180 in human *TIRAP* (top line) and the corresponding amino acid in the murine TIR domain, Serine 200 (bottom line), is highlighted by a red box. **B)** Cloning strategy for the generation of S200L knock-in mice. Targeting vector was constructed covering a sequence from exon 3-7 of murine *Tirap.* This modified sequenced contained a mutated BamH1 site in exon 6 (for use in confirmation of recombination in ES cells by southern blotting and restriction digestion) as well as the mutated coding sequence for murine *Tirap* at codon 200 (TCC (Serine) -> **CT**C (Leucine) (illustrated in **C.**). It also contained the neomycin resistance gene flanked by 2 Flp sites. After confirming homologous recombination occured through southern blotting and digestion of fragments, the neomycin cassette was removed by incubation with Flp recombinase. The resulting DNA was sequenced and annotated sequence for exon 6 is shown in **D**. The resulting S200L mice generated were bred with wild-type mice to generate different combintions of the 3 genotypes annotated as *Tirap*$^{200S/S}$ (wild-type, Serine/Serine homozygotes), *Tirap*$^{200S/L}$ (Serine/Leucine heterozygotes) and *Tirap*$^{200L/L}$ (knock-in, Leucine/Leucine homozygotes), for use in experiments.

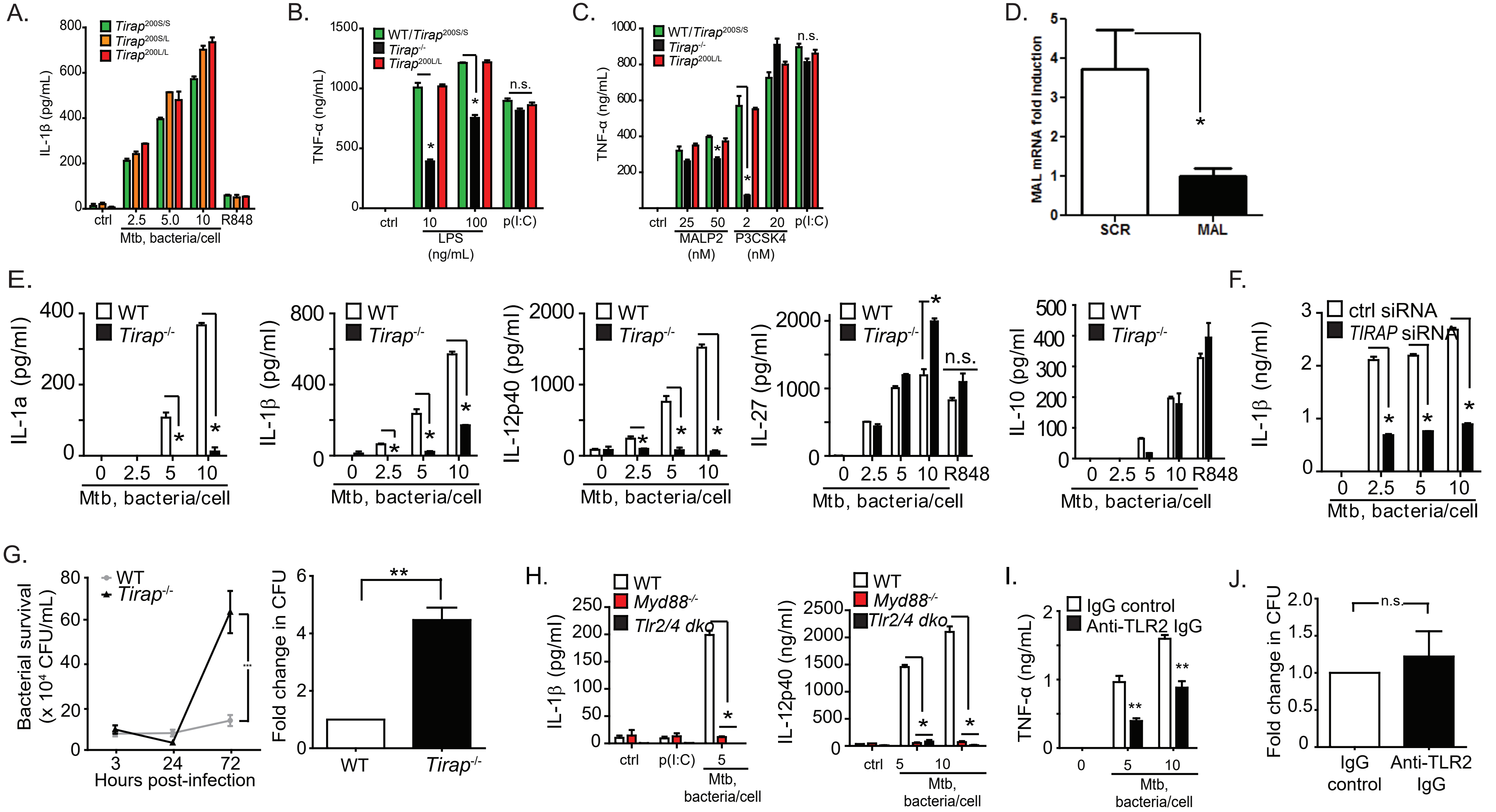

Supplemental Figure 2 - TLR signalling and *Mtb*-induced cytokine production is intact in murine S200L macrophages and Mal is required for cytokine production and killing of intracellular Mtb. (Figures 2 and 3)

(A) IL-1β secretion from primary BMM infected wth *Mtb* H37Rv at the indicated multiplicity of infection was measured in supernatants collected after 20 h stimulation and analysed by ELISA. **(B-C)** TNF-α secretion by primary BMM from wild-type (S/S), homozygote (L/L) and *Tirap*$^{-/-}$ mice stimulated for 20 h with LPS **(B)**, Malp-2, Pam3CysK4 (P3CSK4) or Poly I:C (p(I:C) - 100 ng/ml) **(C)**. All data is mean+/- S.D. from a single experiment (macrophages from 3 mice/group) representative of 3 independent experiments.

(D) confirmation of *TIRAP* knockdown by rtPCR. **(E)** Pro-inflammatory cytokine secretion by primary WT and *Tirap*$^{-/-}$ BMDM infected with Mtb H37Rv. **(F)** IL-1β secretion by THP-1 cells treated with *TIRAP* siRNA or scrambled control. **(G)** Primary murine BMM were infected with Mtb H37Rv and bacterial numbers determined. Data are means +/- S.D of data pooled from 3 separate experiments. **(H)** Pro-inflammatory cytokine secretion by WT, *Myd88*$^{/-}$ and *Tlr2/4* dko iBMM infection with Mtb H37Rv. **(I)** TNFα production by differentiated THP-1 treated with anti-TLR-2 antibody OPN305 or control IgG and infected with Mtb. Supernatants collected after overnight stimulation and analysed by ELISA for all experiments. **(J)** PMA-differentiated THP-1 cells treated with an anti-TLR2 antibody (OPN 305 1μg/ml) were infected with Mtb H37Rv and bacterial numbers determined as above. Data are means +/- S.D of data pooled from 3 separate experiments. * $p < 0.05$ (Mann-Whitney) for all experiments.

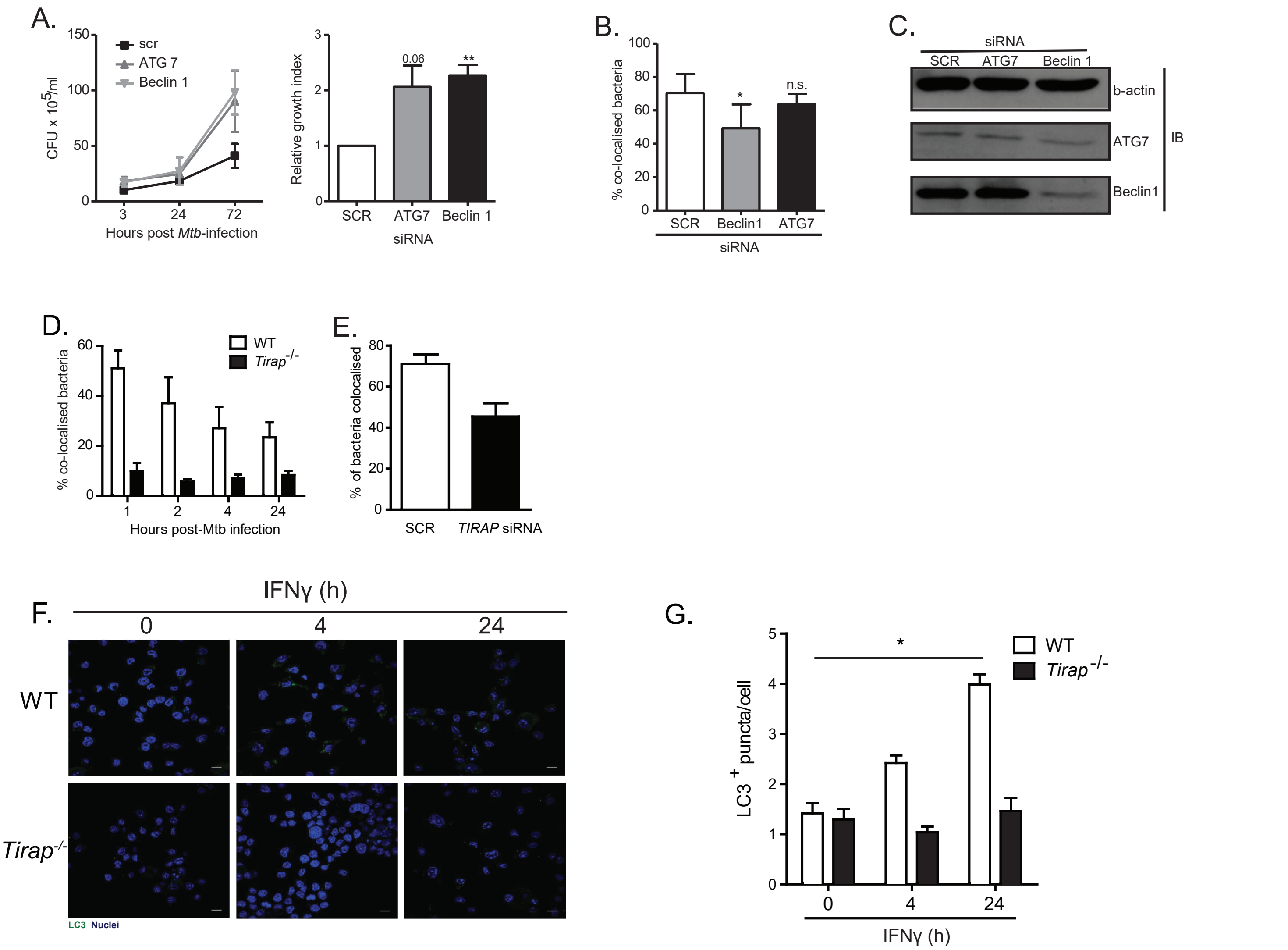

Supplemental Figure 3 - Autophagy deficient macrophages display defects in phagosome maturation and bactericidal activity similar to those seen in Mal-deficient macrophages. (A) WT iBMM were transfected with siRNA against *Beclin 1*, *ATG7* or scrambled control. Transfected iBMM were infected with *Mtb* and lysed at 3, 24 and 72 hours. Lysates were plated out for quantitation of cfus. Representative experiment (left hand panel) and means +/- SD of 3 pooled experiments (right hand panel). **(B)** Transfected iBMM were infected with FITC-labelled Mtb and stained with Lysotracker. Co-localisation was assessed by confocal microscopy. **(C)** Knockdown was assessed by immunoblotting. **(D)** BMDM from WT or *Tirap*$^{-/-}$ mice were infected with FITC-labelled *Mtb* for the indicated times between 0-24 h. Cells were stained with Lysotracker (LT). Co-localisation of *Mtb* with LT+ phagolysosomes was assessed by confocal microscopy. **(E)** THP-1 cells were transfected with siRNA against *Tirap* or scrambled control prior to differentiation with PMA. Cells were were stimulated overnight with recombinant human (rh)IFN-γ (20ng/ml) prior to being infection with FITC-labelled Mtb H37Rv. Cells were fixed at 2 h post-infection and stained with anti-LAMP-1 antibody and a fluorescent secondary antibody. Co-localisation of *Mtb* with LAMP-1+ phagolysosomes was assessed by with confocal microscopy. **(F-G)** Wild-type and *Tirap*$^{-/-}$ immortalised BMM were treated with rmIFN-γ for the time indicated. Cells were fixed, permeabilised and stained with fluorescently labelled anti-LC3 and analysed by confocal microscopy. Representative images shown in (F) and data quantified across 3 independent experiments and analysed by ANOVA (G).

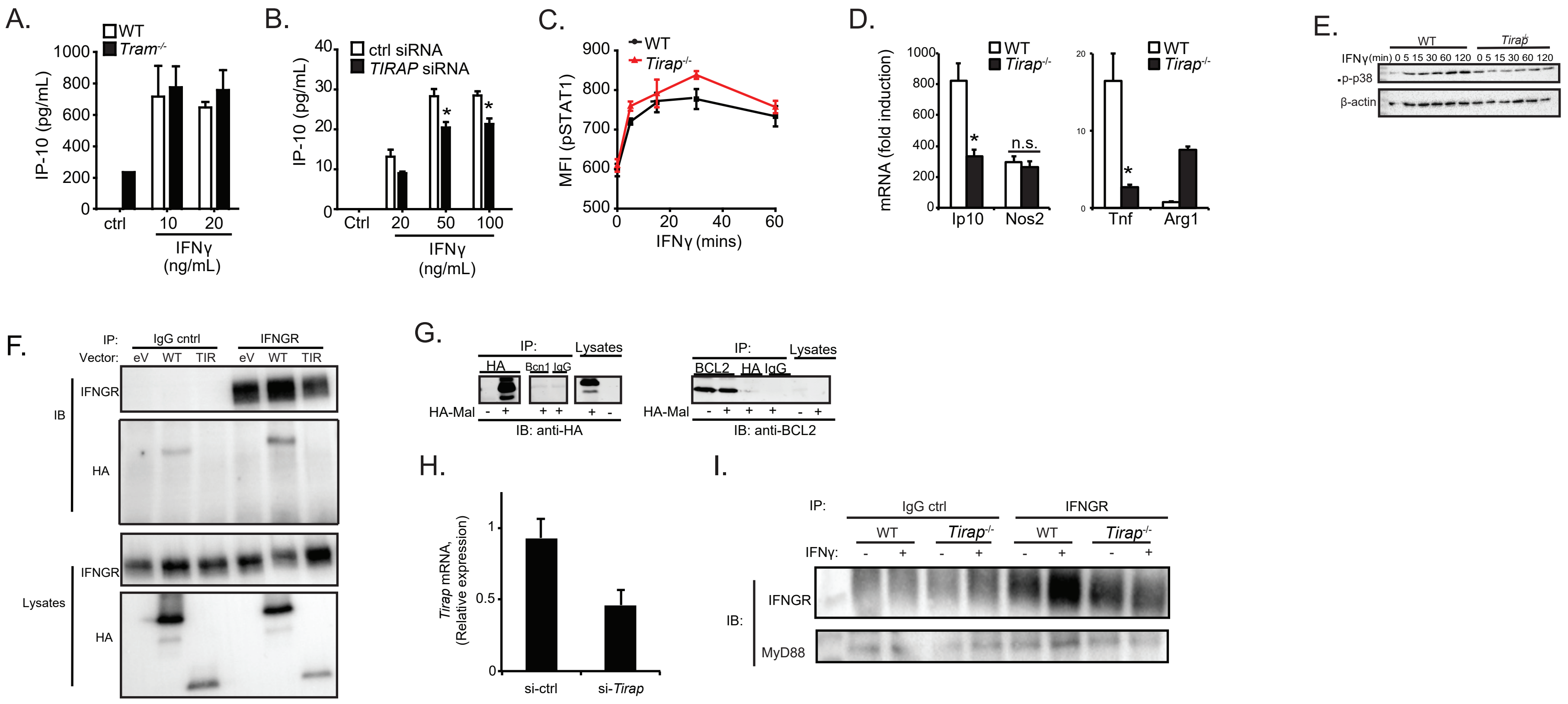

Supplementary Figure 5 - Mal independent processes in macrophage IFNγ signalling **(A)** WT and *Tram* -/- iBMM were treated with rmIFN-γ for 16 hours at the indicated concetrations and supernatants assessed for IP-10 secretion by ELISA. **(B)** THP-1 cells were transfected with siRNA against *TIRAP* or scrambled control prior to differentiation with PMA. Cells were were stimulated overnight with recombinant human (rh)IFN-γ (concentrations indicated) and supernatants analysed for IP-10 by ELISA. **(C)** iBMM were stimulated for the indicated times (0, 5, 15, 30, 60 min) with rmIFN-γ(10 ng/mL) before fixation and staining with anti-pSTAT1 (Y701) antibody conjugated to AlexaFluor 488 before analysis by flow cytometry using a BD FACSCanto II analyzer. **(D)** Primary BMDM (genotype indicated) were treated with recombinant IFN-γ at 20 ng/mL for 4h. RNA was extracted and gene expression analysed using qRT-PCR for the indicated genes. Data is relative fold incution over untreated cells and represents the mean +/- sd for triplicate determinations and is representative of 3 independent experiments. **(E)** Primary WT and *Tirap*-/- BMM were stimulated for for indicated times (0-2 h) with rmIFN-γ (20 ng/mL). Lysates were prepared and analyzed for phosphorylation of p38 MAP-kinase by immunoblotting with anti-p-p38 antibody (Cell Signaling, 9211 - top panel). Blots were stripped and re-probed for β-actin (bottom panel). Data shown are representative of 3 separate experiments. **(F)** HEK-293 cells were transfected with HA-tagged empty vector, HA-Mal or HA-TIR and immunoprecipitation was performed with antibodies to HA (Sigma, H6908) and IFNGR1 (Santa Cruz, sc-700), along with an IgG control, on cell lysates as indicated. Lysates were then blotted with anti-HA antibody. **(G)** HEK-293 cells were transfected with HA-tagged empty vector or HA-Mal and immunoprecipitation was performed with antibodies to HA (Sigma, H6908) and Beclin-1 or BCL-2, along with an IgG control, on cell lysates as indicated. Lysates were then blotted with anti-HA antibody. **(H)** RAW264.7 were transfected with small interfering RNAs specific to mouse *Tirap* (Ambion) for 72h. RNA was extracted and gene expression analysed using qRT-PCR for the indicated genes (*Tirap*) and is presented normalised to Gapdh and relative to untreated/untransfected cells. Data is mean +/- sd for triplicate determinations performed in parallel to the IP experiment shown in Fig 5E. **(I)** Primary WT and *Tirap* -/- BMM were stimulated for 2 h with rmIFN-γ (20 ng/mL) and immunoprecipitation was performed with antibodies to IFNGR1 or an IgG control on cell lysates as indicated. IP-samples were then analyzed for MyD88 expression by immunoblotting with anti-MyD88 (Millipore, 16527), alongside IFNGR1 expression. Data shown for all experiments are representative of 3 independent experiments. .

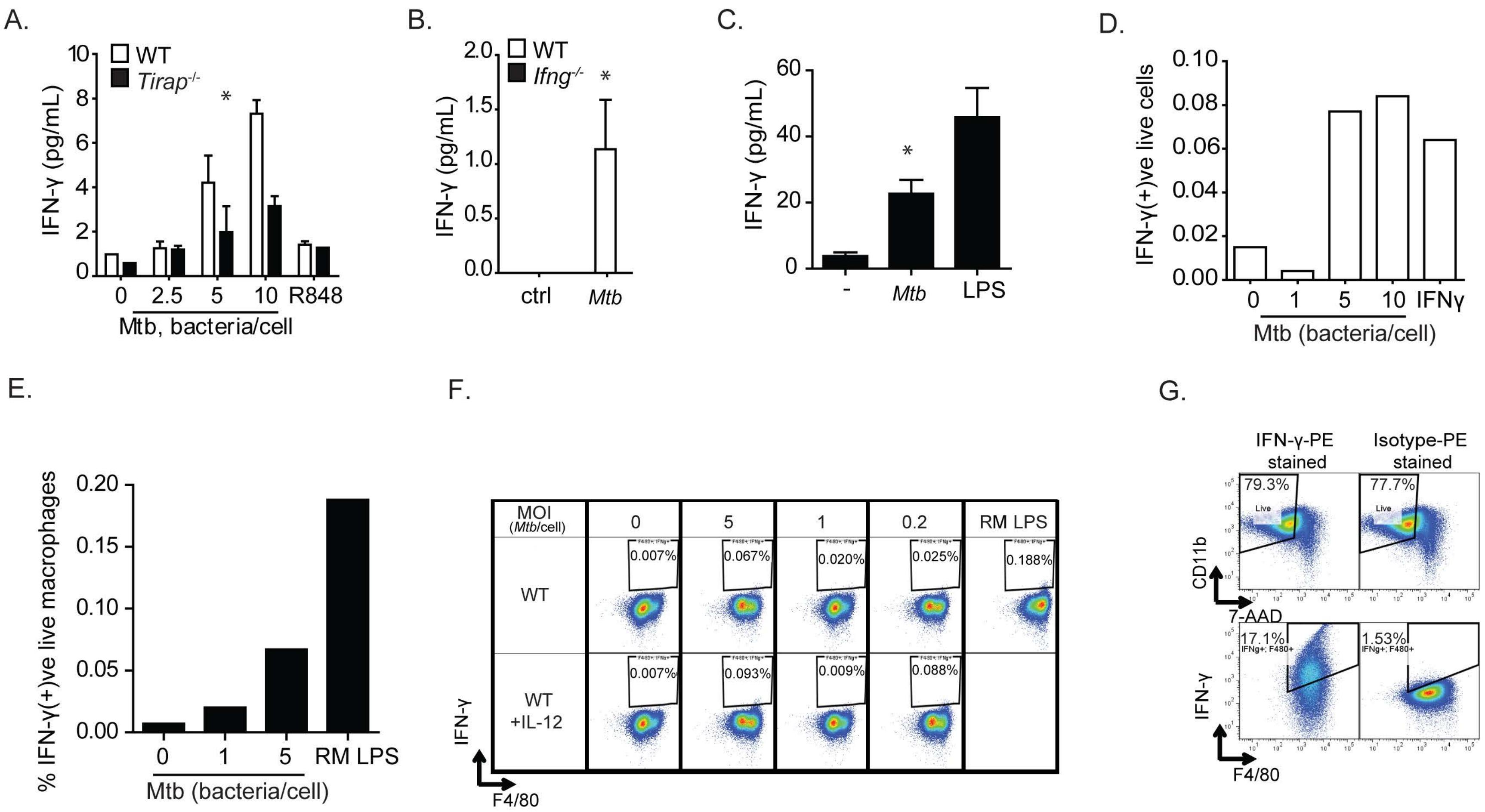

Supplemental Figure 5 - Interferon gamma production in infected macrophages. **(A-C)** Wildtype and *Tirap-/-* immortalized BMM **(A)** or wildtype and *Ifng-/-* primary BMMs **(B)** or PMA differentiated THP1 cells **(C)** were infected with *Mtb*. Supernatants were collected at 48 hours and analyzed for IFNγ production by ELISA. A two way ANOVA was used to compare cell types. Data shown are from a single experiment representative of 3 independent experiments. **(D)** Immortalized WT BMM were infected with *Mtb* for 72 hours. Cells were fixed, permeabilized and stained with live/dead Aqua and fluorescently labeled for the surface markers using anti-CD11b and anti-F4/80, and intracellular anti-IFNγ and analyzed by FACS. Data are expressed as frequency of IFNγ positive cells, gated on aqua negative (live) and CD11b+, F4/80+. Result shown is representative of 3 independent experiments. **(E and F)** Primary WT BMM were pretreated with IL-12 (3ng/ml; WT+IL-12) or medium control (WT) for 24 hours, and then infected with *Mtb* at the multiplicity of infection (MOI) indicated for 24 hours or tolerized with LPS as described in supplemental reference O'Carrol et al, 2013 for 48 hours. Cells were fixed, permeabilized and stained with intracellular fluorescently labeled anti-IFNγ and surface anti-F4/ 80and anti-CD11b and analyzed by FACS. Data representative of 3 independent experiments. **(G)** Cells were tolerized with LPS for 96 hours prior to restimulation with LPS as described in O'Carrol et al, 2013. Cells were fixed, permeabilized and stained with fluorescently labelled anti-IFNγ and anti-CD11b and anti-F4/ 80 and analyzed by FACS to assess intracellular production of IFNγ.

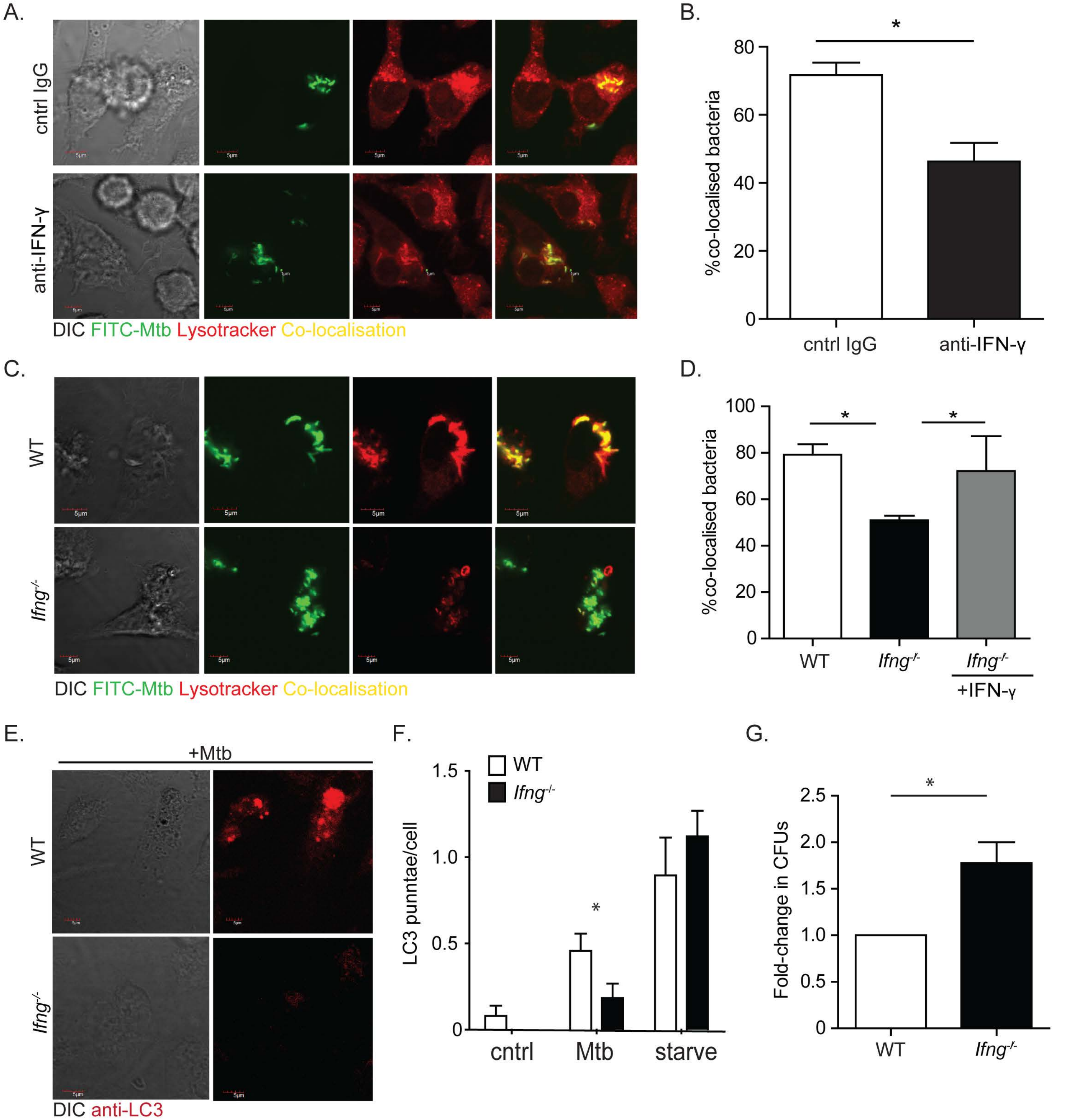

Supplemental Figure 6 - Interferon gamma production by *Mtb* infected macrophages has functional significance. (A and B) Immortalized WT BMM were pretreated with anti-IFNγ antibody or IgG control prior to infection with FITC-labelled *Mtb*. Cells were stained with Lysotracker and fixed. Co-localisation was assessed by confocal microscopy. **(C and D)** Primary WT and *Ifng-/-* BMM with or without pretreatment with IFNγ (20 ng/ml) were infected with FITC-labelled *Mtb*. Cells were stained with Lysotracker and fixed. Co-localisation was assessed by confocal microscopy. **(E and F)** Primary WT and *Ifng-/-* BMM were infected for 16 hours with *Mtb* or treated for 2 hours with starvation medium prior to fixation. Cells were stained with anti-LC3 antibody (Invitrogen) and LC3 puncta/cell were quantified by confocal microscopy. **(G)** Primary WT and *Ifng-/-* BMM were infected with *Mtb* and lysed at 72 h for quantification of bacteria. Experiments show mean +/- SD from a single experiment representative of 3 independent experiments and were analyzed by ANOVA.

Supplemental Experimental Procedures

Cell lines and culture

Primary bone-marrow derived macrophages were derived from the femurs of *Tirap*$^{-/-}$ (Mal knockouts), *Ifng*$^{-/-}$ and wildtype (WT) mice, and differentiated for 7 days with medium containing Macrophage-colony stimulating factor (M-CSF). THP-1 cells (ATCC) were transfected with siRNA against *TIRAP* (Mal) (Dharmacon) and scrambled control using the Amaxa nucleofector and reagents, prior to being differentiated into macrophage-like cells with phorbol myristate acetate (100nmol/L) for 24 h and then cultured in normal medium for a further 48 hours prior to stimulation.

Macrophage stimulation assays

Assessment of bacterial growth

Macrophages were grown at $1x10^5$ cells/ml in 12-well plates in antibiotic-free DMEM supplemented with 10% fetal calf serum. A suspension of *Mtb* H37Rv was prepared as above, and macrophages were infected with *Mtb* at a multiplicity of infection (MOI) of 10 bacteria/macrophage. Extracellular bacteria were washed off at 3 hours post-infection. Cells were lysed at the indicated time points by adding 0.1% Triton-X and scraping. Lysates were centrifuged at 3800 rpm to harvest bacteria, which were plated out at serial ten-fold dilutions on Middlebrook agar, and colonies were counted approximately 21 days later.

Phagosome maturation assays

Macrophages were grown on glass coverslips (Harris et al., 2008) and pretreated with IFN-γ or medium control overnight prior to infection. *Mtb* H37Rv was labeled with FITC (1mg/ml, Sigma) in accordance with the manufacturer's protocol. Bacteria were declumped with glass beads and sonicated prior to being put on to cells. Bacteria were incubated with cells for 1 hour, prior to washing and replacing medium for incubation for a further 1 hour. LysoTracker Red DND-99 (Invitrogen) at 100 nmol/L was added to macrophages one hour prior to fixation. Cells were fixed in 4% paraformaldehyde overnight. Alternatively, for CD63 staining, cells were permeabilized with 0.1% Triton X-100 in PBS after fixation, and blocked with 1% bovine serum albumin and 1% goat serum in PBS for 30 min at room temperature. Cells were incubated with primary antibody (mouse monoclonal antibody against CD63 /LAMP-3; Santa Cruz Biotechnology) at 1μg/mL for 1 h followed by secondary antibody (Alexa Fluor 488– or 568–labelled goat anti–mouse IgG (Invitrogen) were used at 4μg/mL), both at room temperature. Coverslips were mounted onto glass slides with fluorescent mounting medium (Dako), and images were recorded on an Olympus FluoView 1000 and a Zeiss LSM 510 laser scanning confocal microscope. Images were analyzed and prepared using the appropriate proprietary software.

Autophagy Analysis

Autophagosome formation was measured by LC3 punctate staining (Harris et al., 2001). Cells were treated as indicated, fixed using 4% paraformaldehyde, permeabilized using 0.1% Triton-X, blocked with 1% bovine serum albumin and 1% goat serum for 30 minutes at room temperature and incubated with primary antibody (LC3 antibody, Invitrogen) for 1 hour followed by secondary antibody (Alexafluor 488-labelled goat anti-rabbit IgG at 4μg/mL) for one hour. To quantify autophagy, fluorescence microscopy was used to count the number of

LC3- positive punctate dots in primary cells. Each condition was assayed in triplicate, and at least 100 cells per well were counted. Alternatively, LC3 conjugation in cell lysates was evaluated by Western blot analysis using an antibody raised to LC3-I/II (Sigma) used at 1/200, β-actin (Sigma); secondary antibodies (Licor) and chemiluminescence were used for detection. Autophagic flux was inhibited using either bafilomycin (100mM) or a combination of E64d and pepstatin.

Cytokine Measurements

Cytokine measurements were performed in supernatants using commercial ELISA kits from R&D Systems (murine IL-1α, IL-1β, IL-27, IP-10, IFNγ and TNF-α, and human IP-10), eBioscience (human TNF-α and IL-1β), Bioscience (murine IL-6, IL12p40 and IL-10) and Biosera (human IL-10)

Co-Immunoprecipitation

HEK293 cells were incubated for 24 h with DNA encoding various proteins in the presence of Genejuice. Cell lysates were prepared by lysis on ice for 10 min in 50 mM HEPES, pH 7.5, 250 mM NaCl, 20 mM βglycerophosphate, 1% Nonidet-P40, 2 mM dithiothreitol and a protease inhibitor 'cocktail' (Sigma) at a dilution of 1:500 for immunoprecipitation or by being boiled directly in Laemmli sample buffer for immunoblot. Immunoprecipitation was initiated by incubation of lysates for 2 h with protein A/G sepharose beads (Amersham) plus control antibodies. Precleared lysates were then incubated at 4 °C for at least 2 h with various antibodies and protein G beads (Amersham). Washed beads were boiled in sample buffer; proteins were separated by SDS-PAGE and were transferred onto nitrocellulose membrane. Membranes were blotted with the various antibodies and samples were visualized with an enhanced chemiluminescence system (Licor).

Plasmids

HA-Mal has been previously described (Valkov et al., 2011). Site-directed mutagensis was carried out to generate HA-tagged S180L variant Mal which was amplified using Miniprep (Qiagen). The sequences of both HA-Mal and HA-S180L Mal were confirmed by sequencing (Eurofins).

Fluorescence lifetime imaging microscopy-fluorescence resonance energy transfer (FLIM-FRET)

WT and *Tirap*$^{-/-}$ iBMM were treated with IFNγ (10 ng/ml) for 0, 0.5, 1, 2 and 24 h, then fixed in 2% paraformaldehyde and stained with antibodies against IFNGR2 (MyBioSource) and MyD88 (Abcam). For FLIM-FRET studies, they were secondary stained with Alexa Fluor A488 (donor) and A568 (acceptor) antibodies. An Olympus FV1000 microscope equipped with a PicoHarp300 FLIM extension and a 485 nm pulsed laser diode from PicoQuant was used to record FLIM data. Cells were first imaged by confocal microscopy using the Olympus FV1000 system to verify the presence of both donor and acceptor dye. Subsequently, corresponding FLIM images of donor fluorescence were recorded using the PicoHarp extension. Pixel integration time for FLIM images was kept at 40 μs per pixel and fluorescence lifetime histograms were accumulated to at least 10,000 counts in the maximum to ensure sufficient statistics for FLIM-FRET analysis. Photon count rates were kept below 5% of the laser repetition rate to prevent pileup.

Six FLIM images were recorded per time point, with 3 biological repeats per condition and 10 – 30 cells per image in the field of view. FLIM-FRET analysis was performed using the SymPhoTime 64 software (PicoQuant). The fluorescence lifetime decay curve for each image was summed over all pixels, then deconvolved with the measured instrument response

function (IRF) and fitted with a biexponential decay. The amplitude weighted average lifetime was extracted from each fit and averaged over all values of one sample condition. *p* values were determined to assess the significance of donor lifetime changes before and after treatment with IFNγ. A negative control (WT iBMM stained with IFNGR2 + A488 only) and positive control (WT iBMM stained with MYD88 + A488 + A568) were run alongside the samples.

Genotyping

Tirap genotype was determined on DNA extracted from buccal swabs (Isohelix, Cell Products). Ethical permission was granted by the Ethics Committee of the School of Medicine, Trinity College, Dublin. Informed consent was obtained. Genotyping of the Mal S180L and polymorphism was performed using the TaqMan Allelic Discrimination System (PE Biosystems, Foster City, California, USA). Genotyping results were verified using positive sequenced controls. PCR reactions were set up according to the manufacturer's instructions and thermal cycling was performed on 384-well reaction plates on PTC-225 DNA engine Tetrad (MJ Research, San Francisco, California, USA) as follows: initial denaturation and enzyme activation at 95°C for 10 min, followed by 40 cycles of denaturation at 95°C for 15 s and annealing/extension at 60°C for 60 s. Genotypes were determined using an ABI Prism 7900HT (PE Biosystems).

FACS Analysis

Immortalized macrophages were stimulated for the indicated times (0, 5, 15, 30, 60 min) with IFNγ at a final concentration of 10 ng/ml before washing and fixing in 1% PFA. Cells were then permeabilized with Permeabilization Wash Buffer (BioLegend) and stained with anti-pSTAT1 (Y701) antibody conjugated to AlexaFluor 488 at a dilution of 1/50 (Cell Signaling

Technology, clone 58D6) before analysis on a BD FACSCanto II analyzer. For analysis of intracellular IFNγ production primary or immortalized macrophages ($10x10^6$) were infected with the virulent *Mtb* strain at different multiplicities of infection for different times as indicated in the figures. Brefeldin A was added for 4 h at 40 μg/ml in the presence of *Mtb*. Supernatants were removed and detached cells were recovered by centrifugation at 400g for 5 minutes; remaining adherent cells were treated with lidocaine-HCl 4mg/ml supplemented with 10mM EDTA in $Ca2^+/Mg^{2+}$ free PBS for 15 min at 37ºC and detached by vigorous pipetting and finally pooled with the cells recovered previously. To assess purity of the monocultures, cells were stained with a PE-Cy7 labeled anti-mouse F4/80 (eBiosciences) and an APCefluor780 labeled anti-mouse CD11b (eBiosciences) in the presence of Fc block (BD). Dead cells were stained with 7AAD (eBiosciences) for 20 min and fixed with 2% PFA supplemented with actinomycin D (Sigma) at 40μg/ml to prevent background fluorescence due to 7AAD leakage from dead cells. Prior to intracellular staining, cells were permeabilized by incubation in PBS supplemented with 0.1% Saponin+ 0.1% BSA and then stained with a PE-labeled anti-mouse IFN-γ antibody (BD) or corresponding PE-labeled isotype control. *Mtb* was inactivated for 18 h by incubation of the cells in 4% PFA at 4ºC prior acquisition in a FACS Canto II equipped with 488, 633 and 405 lasers. One to two million cells were acquired on the live-cell gate per sample. The gating strategy was as follows: Single cells gated on FSC-H *vs* FCS-A >> Cells gated on FSC-A *vs* SSC-A >> Live (7AADneg), CD11b+ >> F4/80+, IFN-γ+. Gate for IFN-γ positive cells was determined by the corresponding FMO ("fluorescence minus one") control.

For LPS tolerization and recovery, macrophages were treated as previously described in (O'Carrol et al., 2013).

Mice

For *in vivo* experiments, pathogen-free male C57BL/6 (Jackson Laboratory) were maintained under barrier conditions in an animal room at University of Massachusetts Medical School. *Tirap* S200L heterozygote and homozygote mice (C57BL/6 background) were generated as described in Supplemental Figure 1. S200L mice were generated with C57BL/6 embryonic stem cells and C57BL/6 blastocysts. Animals were fed a sterile commercial mouse diet and water ad libitum. The University of Massachusetts Medical School Institutional Animal Care and Use Committee approved these experiments. A sample size for the *in vivo* experiment of 8 animals/group was chosen based on availability of mice. All mice were infected with *Mtb*, so randomisation was not appropriate. Mice were age and sex-matched, and were approimately 4 months old at the experimental endpoint. The investigator measuring the weight of the animals was blinded to the groups, otherwise no blinding was used.

Aliquots of frozen *Mtb* H37Rv were thawed and then sonicated for 5 minutes. A volume of sonicated stock previously titrated to deliver approximately 500 cfu per mouse was added to the nebulizer of a Glas-Col Inhalation Exposure System (Glas-Col, LLC, Terre Haute, IN) and mice were exposed to the infectious aerosol for 30 minutes (Martens et al., 2012). Mice were infected at approximately 8 weeks of age, and were kept in a pathogen-free BSL3 facility for a further 8 weeks. Two mice were killed 24 hours after infection to confirm the actual delivered dose.

Bacterial Load

At 8 weeks, mice were sacrificed. Lung homogenates from six mice were plated to measure bacterial burden. Lungs were homogenized in PBS containing 0.05% Tween-80, diluted serially 10-fold over 4 logs, and plated in duplicate on Middlebrook 7H11 agar (DIFCO,

Becton Dickinson, Sparks, MD). Plates were cultured at 37ºC for 3 weeks and then counted using a dissecting microscope to confirm colony morphology.

Lung Histology

Lungs were inflated and fixed with 10% buffered formalin for 24 hours and then processed for staining. Tissue sections were stained with hematoxylin and eosin (H&E). Sections were made at intervals spanning the whole lung, and the left cranial lobe was examined using light microscopy. Lung surface area of inflammation was measured with a Nikon Eclipse E400 microscope (Nikon Instruments, Melville, NY) at x 20 magnification using Spot Insight v3.5 software (Diagnostic Instruments Inc, Sterling Heights, MN). Percent total lung area involved with inflammation was calculated by dividing the cumulative area of inflammation by the total lung surface area examined for each lung studied.

Lung cytokine expression

Lungs were homogenized in PBS-T, an equal volume of cell lysis buffer (0.5% Triton X-100, 150 nM NaCl, 15 mM Tris, 1 mM $CaCl_2$ and 1mM $MgCl_2$, pH 7.4) was added and the mixture was vortexed, incubates (20 min, 4ºC), vortexed again, centrifuged (10 min, 12,000-14,000 x *g*) and the supernatant was filter-sterilized. Lung lysates were assayed for TNFα by ELISA (R&D Systems).

Statistical Analysis

A one-way ANOVA was performed to assess for statistically significant difference of the means between groups. Chi-squared analysis was used to assess statistically significant proportions of co-localization between groups. P values <0.05 were considered significant. Error bars represent standard deviations of the mean.

References

Harris, J., Hope, J.C., and Lavelle, E.C. (2009). Autophagy and the Immune Response to TB. Transboundary and Emerging Diseases 56 , 248-254.

O'Carroll, C., Fagan, A., Shanahan, F., Carmody, R. (2013). Identification of a Unique Hybrid Macrophage-Polarization State following Recovery from Lipopolysaccharide Tolerance. J. Immunol 192, 427-436.

Valkov, E., Stamp, A., Dimaio, F., Baker, D., Verstak, B., Roversi, P., Kellie, S., Sweet, M.J., Mansell, A., Gay, N.J., *et al.* (2011). Crystal structure of Toll-like receptor adaptor MAL/TIRAP reveals the molecular basis for signal transduction and disease protection. Proc Natl Acad Sci U S A *108*, 14879-14884.
